# Gut microbiome in the Graves’ disease: Comparison before and after anti-thyroid drug treatment

**DOI:** 10.1371/journal.pone.0300678

**Published:** 2024-05-31

**Authors:** Chaiho Jeong, Hansang Baek, Jaewoong Bae, Nakwon Hwang, Jeonghoon Ha, Young-Seok Cho, Dong-Jun Lim

**Affiliations:** 1 Division of Endocrinology and Metabolism, Department of Internal Medicine, College of Medicine, The Catholic University of Korea, Seoul, Republic of Korea; 2 R&D Institute, BioEleven Co., Ltd., Seoul, Republic of Korea; 3 Division of Gastroenterology, Department of Internal Medicine, College of Medicine, The Catholic University of Korea, Seoul, Republic of Korea; University of California Los Angeles, UNITED STATES

## Abstract

While several studies have proposed a connection between the gut microbiome and the pathogenesis of Graves’s disease (GD), there has been a lack of reports on alteration in microbiome following using anti-thyroid drug treatment (ATD) to treat GD. Stool samples were collected from newly diagnosed GD patients provided at baseline and after 6 months of ATD treatment. The analysis focused on investigating the association between the changes in the gut microbiome and parameter including thyroid function, thyroid-related antibodies, and the symptom used to assess hyperthyroidism before and after treatment. A healthy control (HC) group consisting of data from 230 healthy subjects (110 males and 120 females) sourced from the open EMBL Nucleotide Sequence Database was included. Twenty-nine GD patients (14 males and 15 females) were enrolled. The analysis revealed a significant reduction of alpha diversity in GD patients. However, after ATD treatment, alpha diversity exhibited a significant increase, restored to levels comparable to the HC levels. Additionally, GD patients displayed lower levels of Firmicutes and higher levels of Bacteroidota. Following treatment, there was an increased in Firmicutes and a decrease in Bacteroidota, resembling levels found in the HC levels. The symptoms of hyperthyroidism were negatively associated with Firmicutes and positively associated with Bacteroidota. GD had significantly lower levels of *Roseburia*, *Lachnospiraceaea*, *Sutterella*, *Escherichia-shigella*, *Parasuterella*, *Akkermansia*, and *Phascolarctobacterium* compared to HC (all p < 0.05). Post-treatment, *Subdoligranulum* increased (p = 0.010), while *Veillonella* and *Christensenellaceaea R-7 group* decreased (p = 0.023, p = 0.029, respectively). *Anaerostipes* showed a significant association with both higher smoking pack years and TSHR-Ab levels, with greater abundantce observed in smokers among GD (p = 0.16). Although reduced ratio of Firmicutes/Bacteroidetes was evident in GD, this ratio recovered after treatment. This study postulates the involvement of the gut microbiome in the pathogenesis of GD, suggesting potential restoration after treatment.

## Introduction

‘Gut microbiota’ is a term that collectively refers to the bacteria, archaea, and eukarya colonizing the gastrointestinal tract. Accumulated studies have shown that regulation and alterations of the gut microbiome are associated with a variety of disorders, such as type II diabetes, asthma, obesity, autism spectrum disorders, and thyroid cancer [1–5]. Recent studies have emphasized the role of the gut microbiome in the pathogenesis of autoimmune diseases such as rheumatoid arthritis, systemic lupus erythematosus, Hashimoto’s thyroiditis, and Graves’ disease (GD) [6–9]. GD induces hyperthyroidism in which thyroid hormone receptor stimulating antibodies (TSHR-Ab) mimic the function of thyroid stimulating hormone (TSH) to activate the thyroid. However, the pathogenesis of GD has yet to be fully elucidated, and the mechanisms triggering the autoimmune attack on the thyroid remain unknown.

The gut microbiome are considered to play an essential role in host immune homeostasis and the release of metabolites developing a diseased condition [10]. A change in the gut microbiome impact the permeability of the intestinal mucosal barrier, which may lead to increase in toxins, antigens, bacterial metabolites, and can even facilitated the entry of bacteria into the bloodstream from the intestine, triggering inflammation. This could act as trigger for autoimmunity, potentially leading to the development of cause autoimmune diseases [11]. Therefore, several studies have sought to elucidate the roles played by the gut microbiome in the pathogenesis of GD [12, 13]. Notably, experiment involving fecal microbiota transplantation from GD patients have demonstrated in the severity of GD features in mouse models [14].

The predominant therapy for GD in Europe, Asia, and the USA involve the use of an anti-thyroid drug (ATD) [15]. Nevertheless, there is a lack of reports regarding in the alterations in microbiome after treatment of GD with the ATD. Therefore, we used next-generation-based sequencing to analyze the change in gut microbiome among GD patients after 6 months of ATD treatment. Our findings not only show the gut microbiome profile of GD patients but also provide substantial data on the changes in gut microbiome post-treatment. This sheds lights on the causal relationship in GD and introduces novel insights for treatment of the disease. Additionally, we conducted an analysis to explore the association between the modification of the gut microbiome and thyroid function along with antibodies.

## Materials and methods

### Participants

The participants were recruited between May 2020 to December 2021 from the Division of Endocrinology and Metabolism, Seoul St. Mary’s Hospital, Korea. (ClinicalTrials.gov number: NCT04383795). Informed written consent was obtained from all study participants. GD patients were initially diagnosed following the 2016 American Thyroid Association Guidelines for the diagnosis and management of hyperthyroidism and other causes of thyrotoxicosis [16]. Ultimately, 29 GD patients, comprising 14 males and 15 females, were enrolled. For the control group, fecal microbiota data from 230 healthy subjects (110 males and 120 females) were included. The data of the healthy controls were sourced from an open public project named ‘Fecal microbiota in the Korean population’ of The European Molecular Biology Laboratory (EMBL) Nucleotide Sequence Database (freely available at EMBL-EBI under accession number PRJEB35019) [17]. This public data was generated by our co-author at R&D Institute, BioEleven Co., Ltd). Consequently, the same sequencing method was used. The healthy control group proclaimed the absence of any specific disease through a self-reported questionnaire.

Newly diagnosed Graves’ disease (GD) patients provided stool samples both at the beginning of the study and after 6 months of treatment with antithyroid drugs (ATD), including methimazole, carbimazole, and propylthiouracil. The administration of ATD was integral to the hospital care, with the specific choice of ATD being determined by the physician’s preference and discretion. Four patients did not accomplish the second sampling (one transferred to another medical center, one moved abroad, and two were lost to follow-up). None of the GD patients and normal healthy subjects had documented history of gastrointestinal illness, nor had they used prebiotics, probiotics, and antibiotics in the two months preceding sampling. The exclusion criteria included: 1) inability or refusal to provide written informed consent, 2) current use of drugs that may affect intestinal flora such as probiotics/prebiotics, 3) pregnancy, and 4) presence of acute infectious disease, inflammatory bowel disease (IBD), or malignancy. The authors ensured that information identifying individual participants was accessible only during data collection. This study adhered to the tenets of the Declaration of Helsinki and was approved by the Institutional Review Board of Catholic Medical Center (No KC19TESI0523). Informed written consent was obtained from all study participants.

### Sample collection

All fecal samples were collected in an icebox within 1 hour of defecation, then stored at -80° until DNA extraction. A questionnaire was filled out regarding dietary habits, age, gender, body weight, smoking, and health condition. Additionally, a symptom rating scale (SRS) for assessing hyperthyroidism was administered to GD patients before and after ATD treatment. SRS is an easily administered observer-rated scale known for its high interrater reliability, providing an effective assessment of symptom severity and therapy response (S1 Table) [18].

Several laboratory parameters were measured at baseline and after treatment. These included thyroid receptor antibodies (TSH-R Ab <1.5), TSH (0.27–4.2 μIU/ml), T3 (80–200 ng/dL), Free T4 (0.93–1.7 ng/dL), anti-TG Ab (< 115 IU/mL), and anti-TPO Ab (<34 IU/ml).

### DNA extraction

The total genomic DNA extraction was performed from 200 mg of stool sample using a Maxwell® RSC PureFood GMO and Authentication Kit (Promega, Madison, WI) according to the manufacturer’s instructions. The DNA concentration were measured by a UV-vis spectrophotometer (NanoDrop 2000c, Wilmington, DE) and quantified by a QuantiFluor® ONE dsDNA System (Promega). All extracted DNA samples were stored at –20°C until used for further experiments.

### PCR amplification of the V3 –V4 regions of the bacterial 16S rRNA gene

The V3-V4 region in the bacterial 16S rRNA gene was amplified using primer sets F319 (5′- TCGTCGGCAGCGTCAGATGTGTATAAGAGACAGCCTACGGGNGGCWGCAG) and R806 (5′- GTCTCGTGGGCTCGGAGATGTGTATAAGAGACAGGACTACHVGGGTATC-TAATCC– 3′). The amplified products were purified using AMpure XP magnetic beads (Beckman Coulter Inc.) according to the manufacturer’s protocol. The purified products were then quantified using a QuantiFluor® ONE dsDNA System (Promega). The product size and quality were evaluated by Bioanalyzer 2100 (Agilent). Finally, the pooled libraries were sequenced using an Illumina MiSeq instrument with a MiSeq v3 Reagent Kit (Illumina Inc.).

### Gut microbiome analysis

The gut mcirobiota analysis were performed with QIIME 2 [19]. Demultiplexed sequence data using MiSeq Reporter were joined using the q2-vsearch plugin. Sequences were quality filtered using the q2-quality-filter plugin followed by denoising with Deblur [20] (via q2‐deblur). All amplicon sequence variants (ASVs) were aligned with mafft [21] (via q2‐alignment). Alpha‐diversity metrics (Chao1, Shannon and Simpson’s diversity index) were estimated using q2‐diversity after samples were rarefied with depth of 6525 (subsampled without replacement). Taxonomy was assigned to ASVs using the q2‐feature‐classifier [22] classify‐sklearn naive Bayes taxonomy classifier against the SILVA database [23]. Non-metric multidimensional scaling (NMDS) plot was performed using phyloseq and ggplot2 and the dissimilarity was based on Bray Curtis distance. Analysis of molecular variance (AMOVA) (ade4 package in R) was used to determine the significant differences in bacterial structure [24]. We carried out linear discriminant analysis effect size (LEfSe) analysis to detect significant differences in bacterial taxonomonies (LDA score > 3.0). The top nine microbiota were selected to serve as biomarkers (LDA score > 3.0) for the diagnosis of GD. We conducted the 5-fold cross-validation and generated receiver-operating characteristic (ROC) curves and area under the curve for a logistic model. Spearman correlation coefficients were used to analyze the correlation between gut microbiota and serum laboratory findings and SRS. Gut microbiota data from GD patients before and after ATD treatment was used for correlation analysis.

## Results

The clinical characteristics of the study participants, both before and after ATD treatment, are listed in Table 1. The participants, aged between 20 and 68 years, had a mean age of 45.7 ± 12.3 years. The average body mass index was 23.2 ± 3.7 kg/m^2^. Fifteen participants (51.7%) were female and seven of them had experienced menopause. Treatment details revealed that sixteen participants received methimazole (10–30 mg), twelve were treated with carbimazole (10–30 mg), and one was administered propylthiouracil (100mg). Throughout the follow-up period, the doses of these ATD were adjusted based on individual patient’s clinical symptoms and laboratory findings. There was no significant difference in microbiota with a relative abundance greater than 1% between patients treated with methimazole and those treated with carbimazole. The average SRS was 13.8 ± 8.2 points. Among the participants, 51.7% were current or past smokers, with an average pack-years of 18.7 ± 29.4. Baseline level of free T4, TSH, and TSHR-Ab levels were 4.5 ± 2.6 ng/dL, 0.01 ± 0.01 uIU/mL, and 9.5 ± 8.0 IU/L, respectively. After 6 months, these levels changed to 1.3 ± 0.6 ng/dL, 2.5 ± 3.6 uIU/mL, and 6.1 ± 6.8 IU/L, respectively.

**Table 1:** Clinical characteristics of the study participants with Graves’ disease.

Total	Baseline (n = 29)	After 6 months (n = 25)	reference
Age (years)	45.7 ± 12.		
Sex, n			
Male	14	13	
Female	15	12	
Body mass index (kg/m^2^)	23.2 ± 3.7	24.5 ± 3.8	
Symptom score	13.8 ± 8.2	5.4 ± 7.6	
Smoking			
Current smoker, n (%)	7 (24.1%)		
Past smoker, n (%)	8 (27.6%)		
Pack years	18.7 ± 29		
Family history of hyperthyroidism, n (%)	7 (24.1%)		
Anti-thyroid drug, n (%)			
Methimazole	16 (55.2%)		
Carbimazole	12 (44.8%)		
Propilthiouracil	1		
TSH (uIU/mL)	0.01 ± 0.01	2.5 ± 3.6	0.55–4.78
Free T4 (ng/dL)	4.5 ± 2.6	1.3 ± 0.6	0.89–1.76
T3 (ng/mL)	3.9 ± 2.0	1.4 ± 0.5	0.6–1.81
TSH receptor Antibody (IU/L)	9.5 ± 8.0	6.1 ± 6.8	< 1.75
TSI bioassay (%)	529.4 ± 190.1	348.5 ± 201.2	< 140
TPO Antibody (IU/mL) (	1503.2 ± 2504.8		< 60
Thyroglobulin Antibody (IU/mL)	345.5 ± 543.9		< 60
Systolic blood pressure (mmHg)	123.6 ± 16.8	122.5 ± 11.3	
Diastolic blood pressure (mmHg)	76.0 ± 10.5	78.3 ± 10.4	

TSH; thyroid stimulating hormone, TSI; thyroid stimulating immunoglobulin; TPO; thyroid peroxidase

### Difference in gut microbiota between GD patients and healthy controls

**Fig 1:**
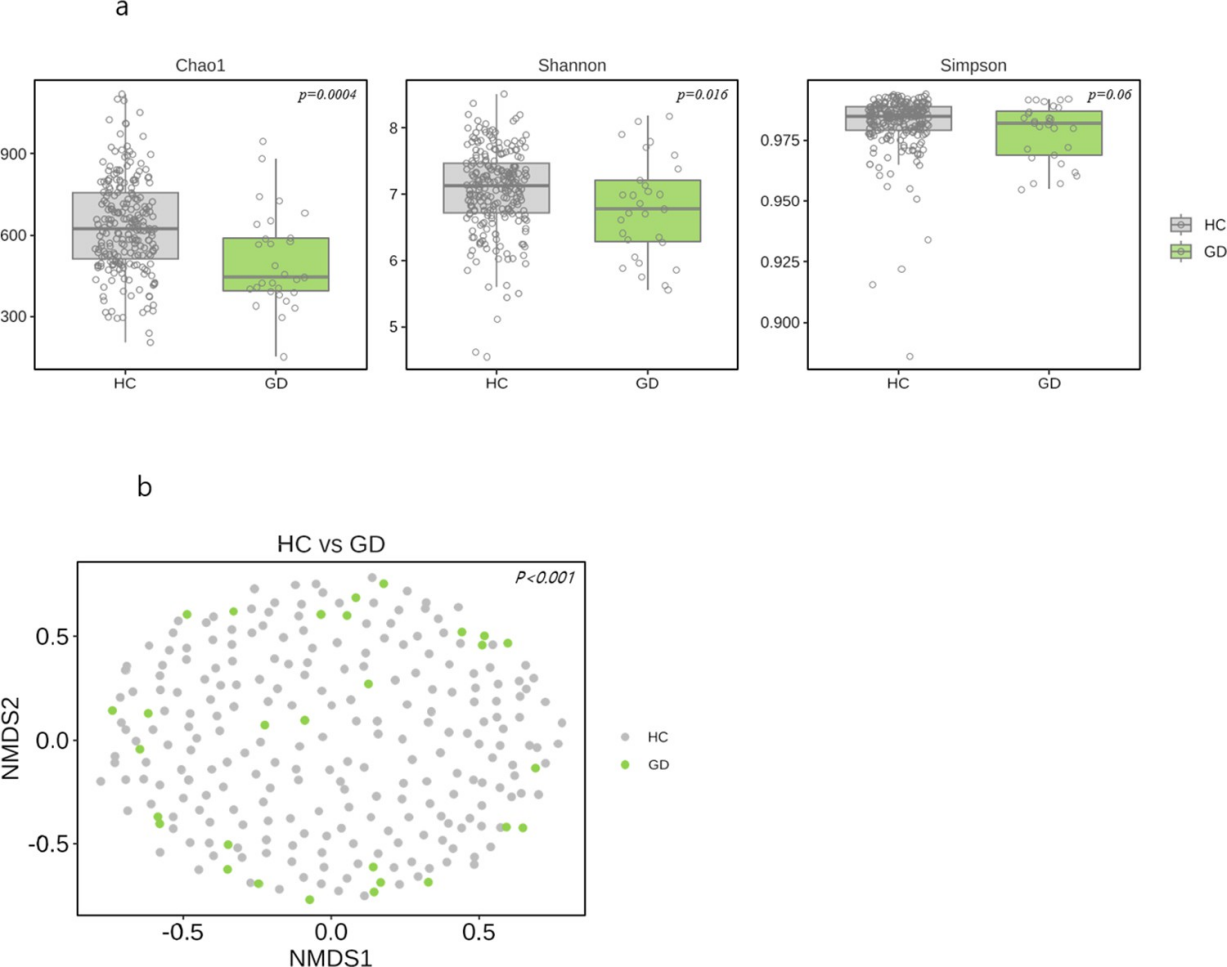
(a) Alpha diversity between GD and HC. (b) Beta diversity between GD and HC. GD, Graves’ disease; HC, healthy control; NMDS, non-metric multidimensional scaling.

Fig 1 shows the difference in alpha diversity between 29 GD patients and the 230 healthy control (HC) group. In GD patients, the alpha diversity, as measured by Chao1, Shannon, and Simpson indexes, was consistently lower (p-values: 0.0004, 0.016, and 0.06, respectively). Additionally, there was significant dissimilarity in beta diversity between the GD and HC groups (p < 0.001) (Fig 1).

#### 1) Difference in gut microbiota at phylum level

**Fig 2:**
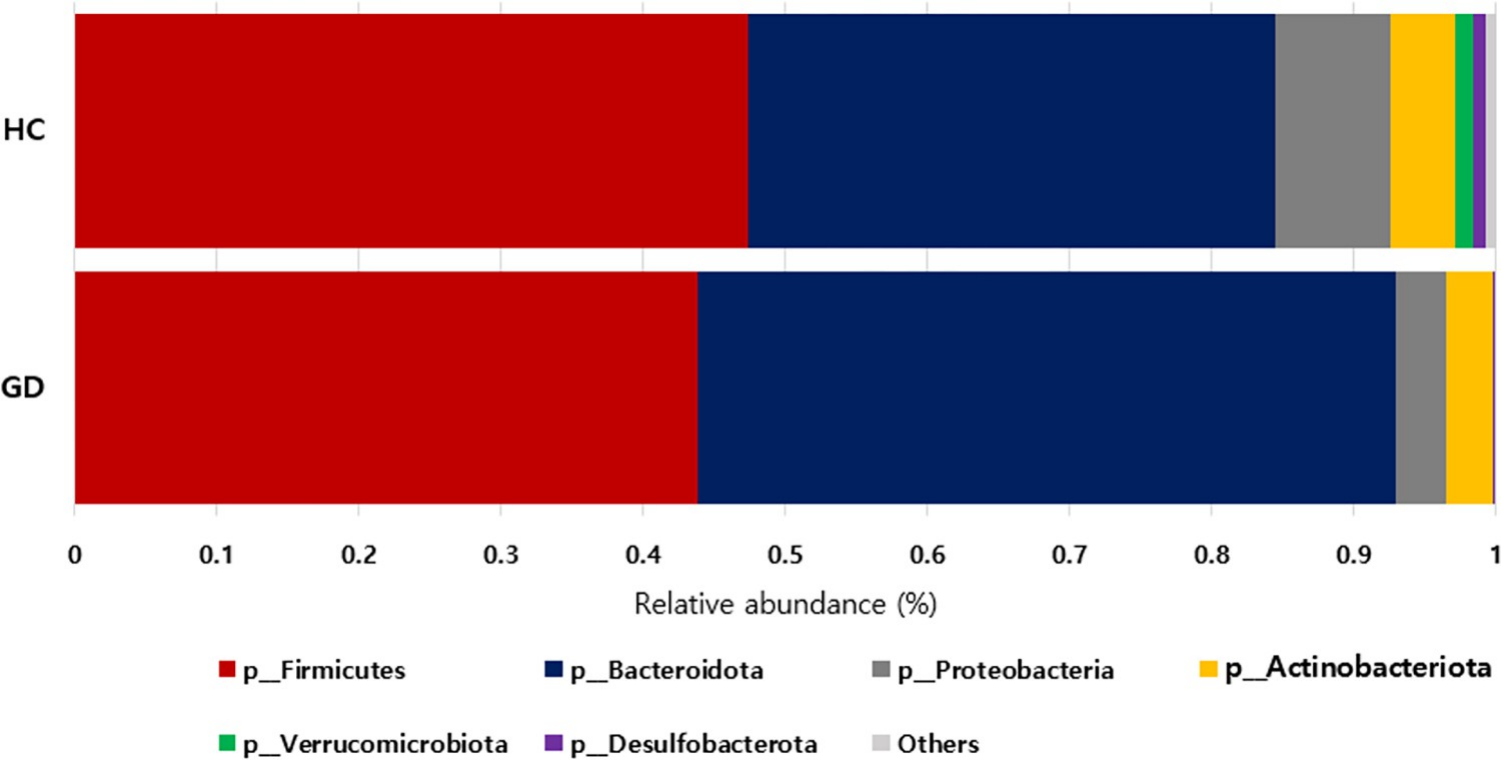
Taxonomy composition in GD patients (n = 29) and HC (n = 130) at phylum level. GD, Graves’ disease; HC, healthy control.

**Fig 3:**
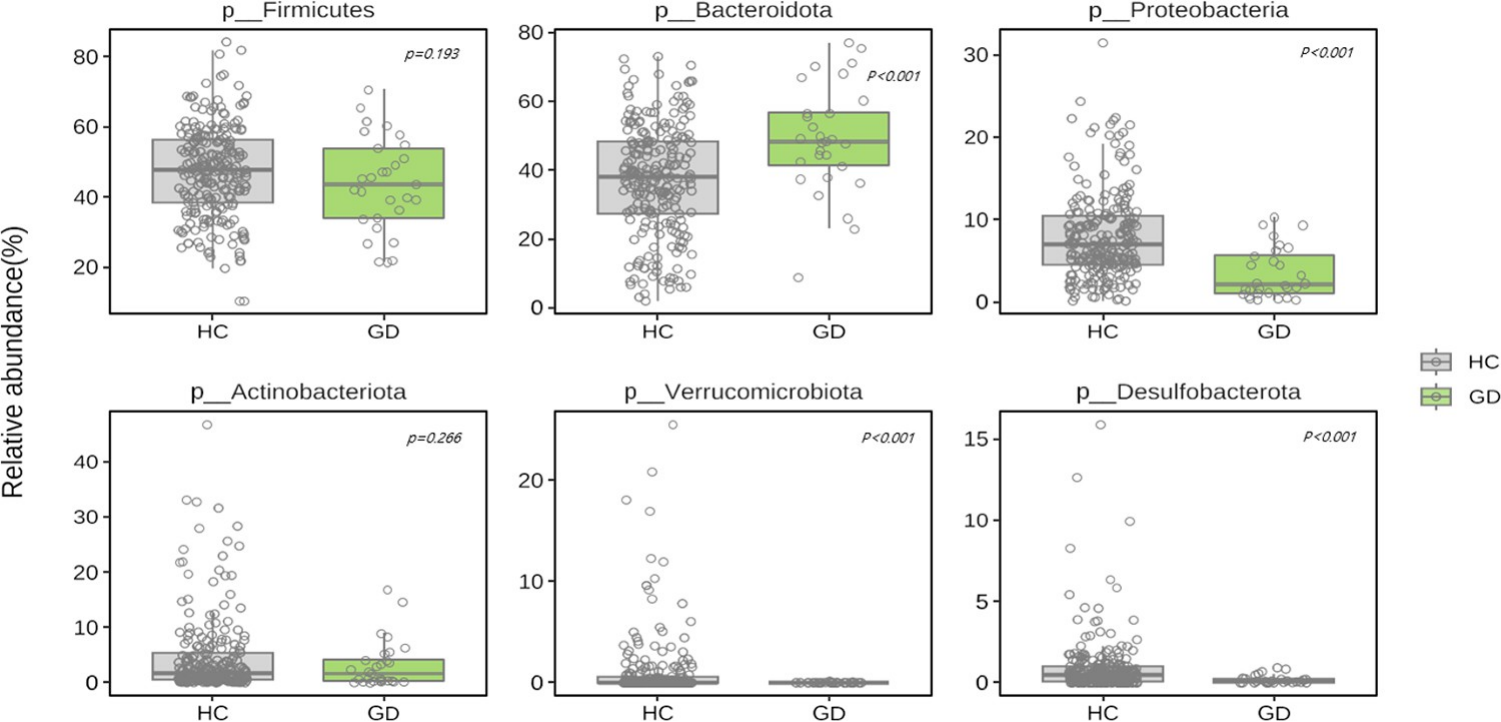
Differences in bacterial phyla between GD patients (n = 29) and HC (n = 130). GD, Graves’ disease; HC, healthy control.

**Fig 4:**
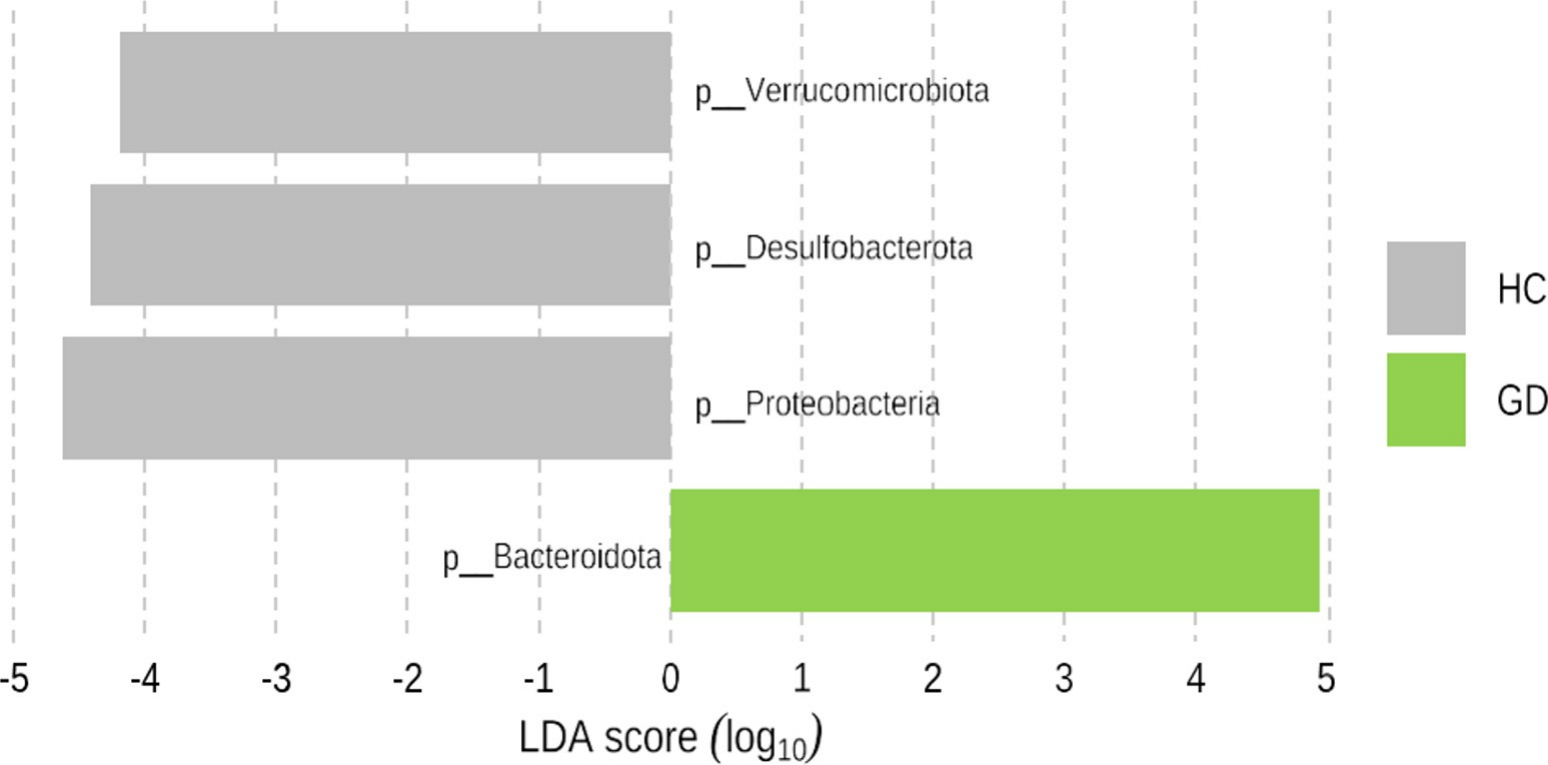
Distinguishment in bacterial phyla between GD patients (n = 29) and HC (n = 130) according to LEfSe algorithm. GD, Graves’ disease; HC, healthy control.

Fig 2 and S2 Table shows the taxonomic composition in GD and HC groups at the phylum level. Among microbiota with more than 1% in relative abundance, GD patients showed lower levels of Firmicutes (p = 0.193) and significantly higher levels of Bacteroidota (p < 0.001) compared to HC. Additionally, GD patients showed lower abundance of Proteobacteria, Verrucomicrobiota, and Desulfobacterota (all p < 0.001; Figs 3 and 4). The relative abundance of Firmicutes/Bacteroidota was lower in GD patients (1.2 ± 1.3) compared to the HC group (2.2 ± 3.1; p = 0.003).

#### 2) Difference in gut microbiota at genus level

**Fig 5:**
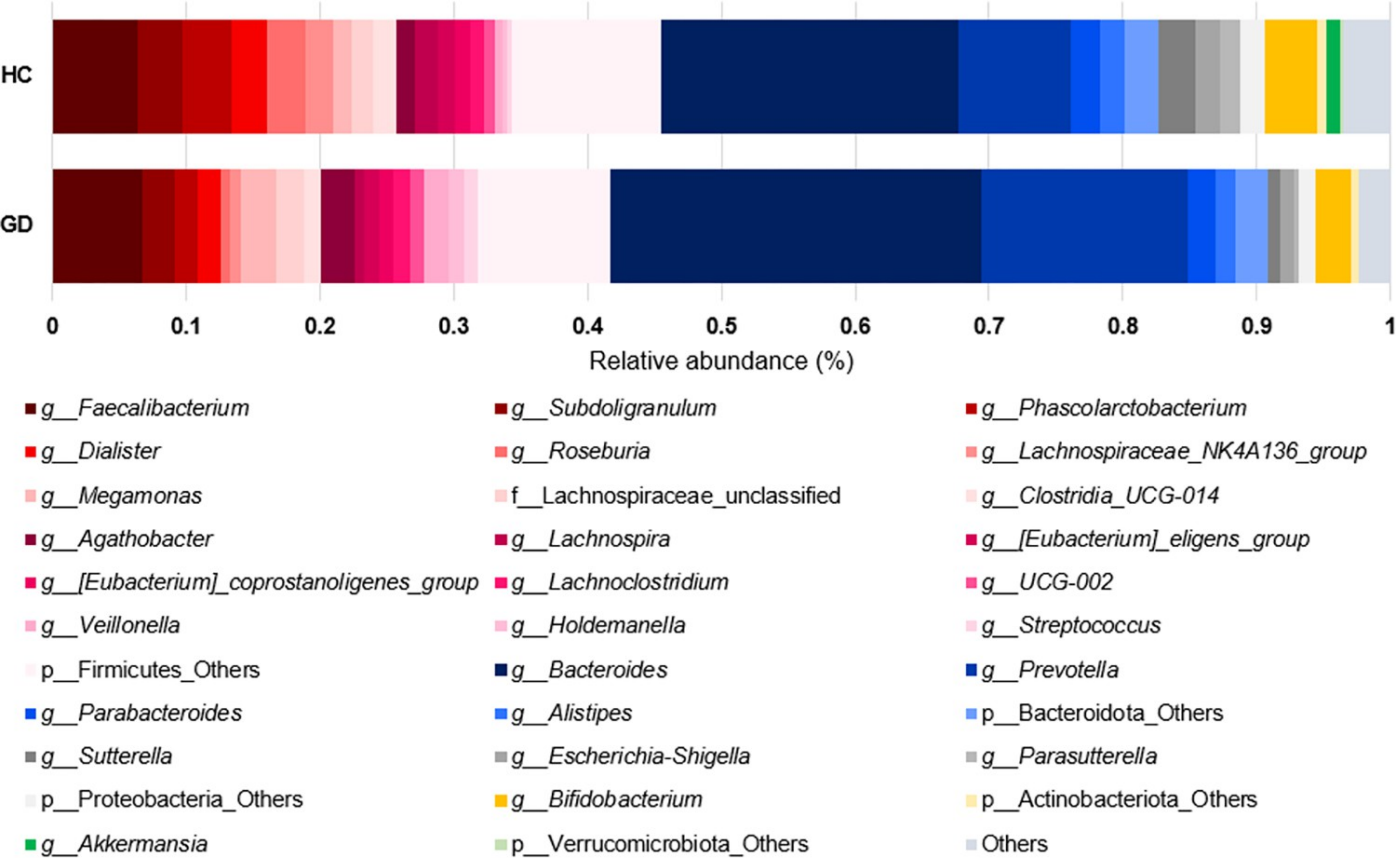
Taxonomy composition in GD patients (n = 29) and HC (n = 130) at genus level. GD, Graves’ disease; HC, healthy control.

**Fig 6:**
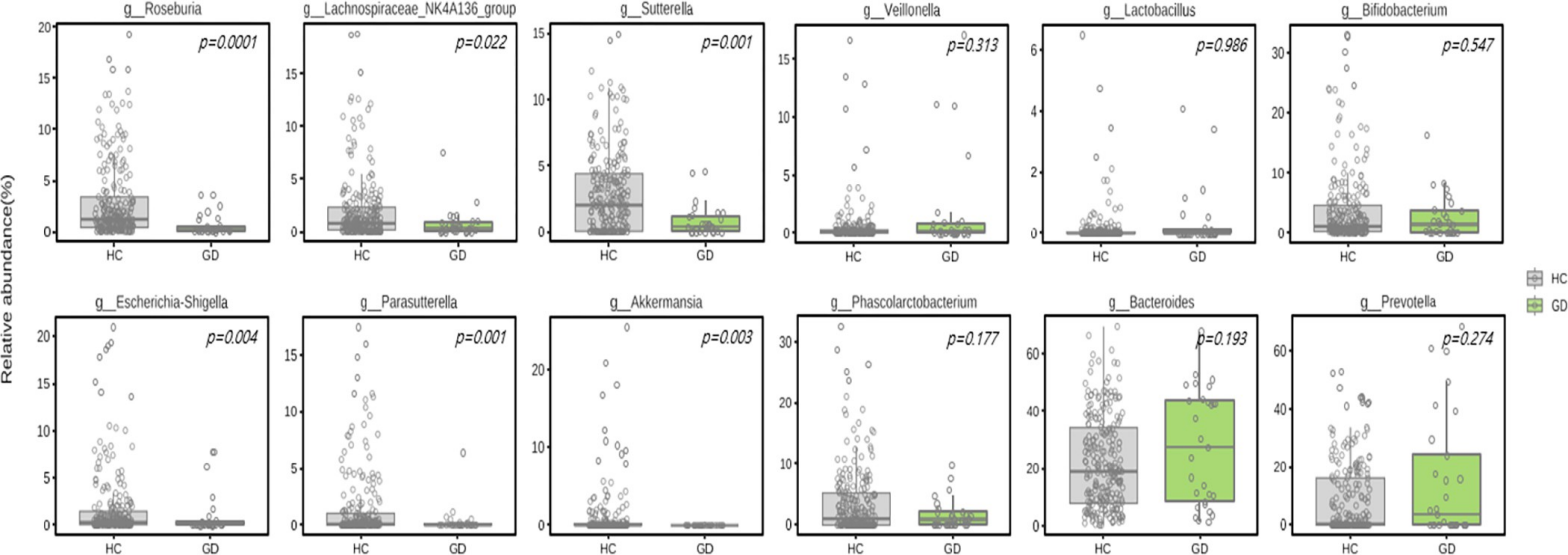
Differences in bacterial genus between GD patients (n = 29) and HC (n = 130) at genus level. GD, Graves’ disease; HC, healthy control.

**Fig 7:**
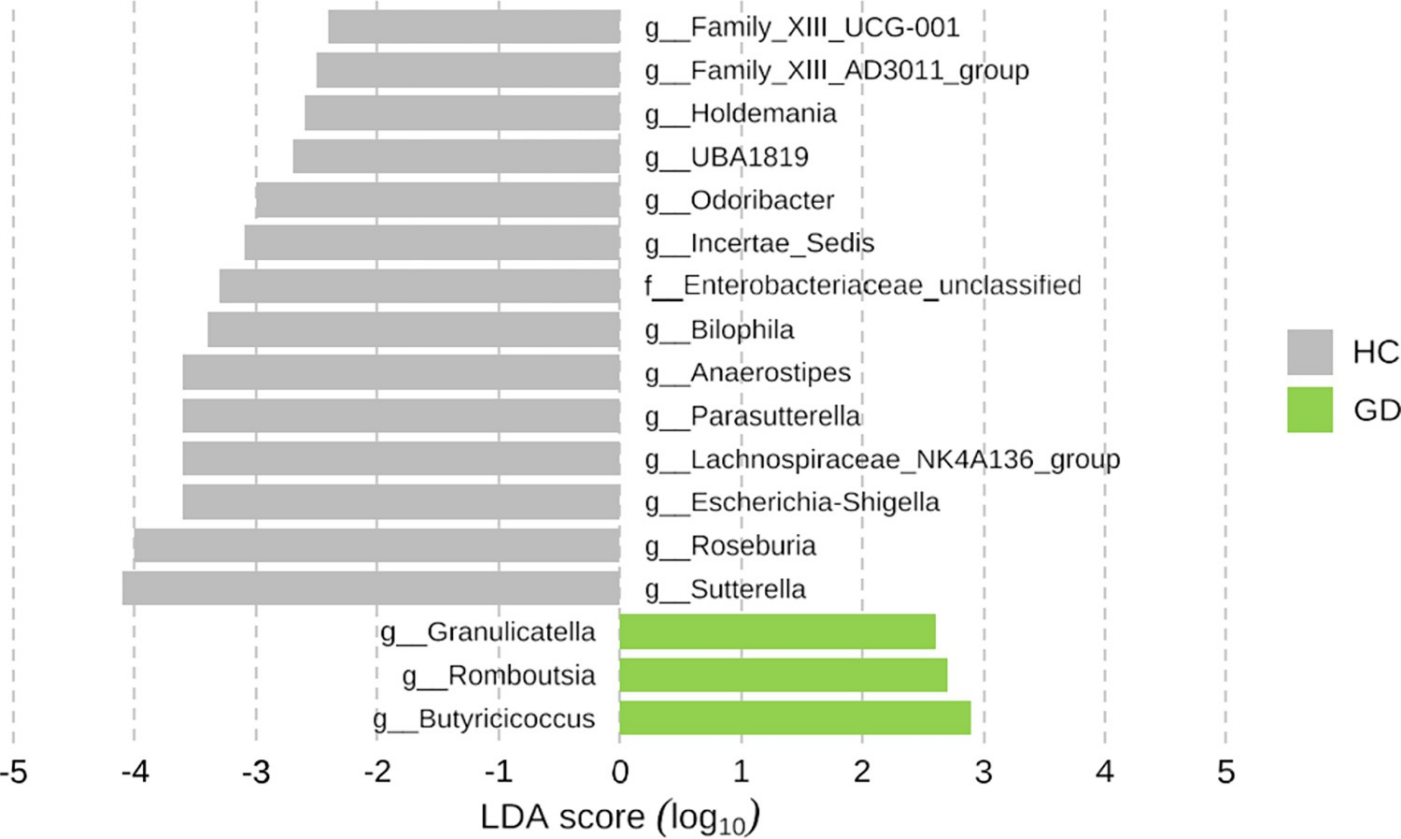
Distinguishment in bacterial genus between GD patients (n = 29) and HC (n = 130) according to LEfSe algorithm. Graves’ disease; HC, healthy control.

Fig 5 and S3 Table shows the taxonomy composition at the genus level in GD and HC groups. GD showed significantly lower levels of *Roseburia*, *Lachnospiraceaea*, *Sutterella*, *Escherichia-shigella*, *Parasuterella*, *Akkermansia*, and *Phascolarctobacterium* than HC (all p < 0.05; Fig 6). Additionally, using a LEfSe algorithm, we identified 9 genera with significant distinctions between the two groups (LDA score > 3.0; Fig 7).

#### 3) ROC curve analysis to diagnose GD

**Fig 8:**
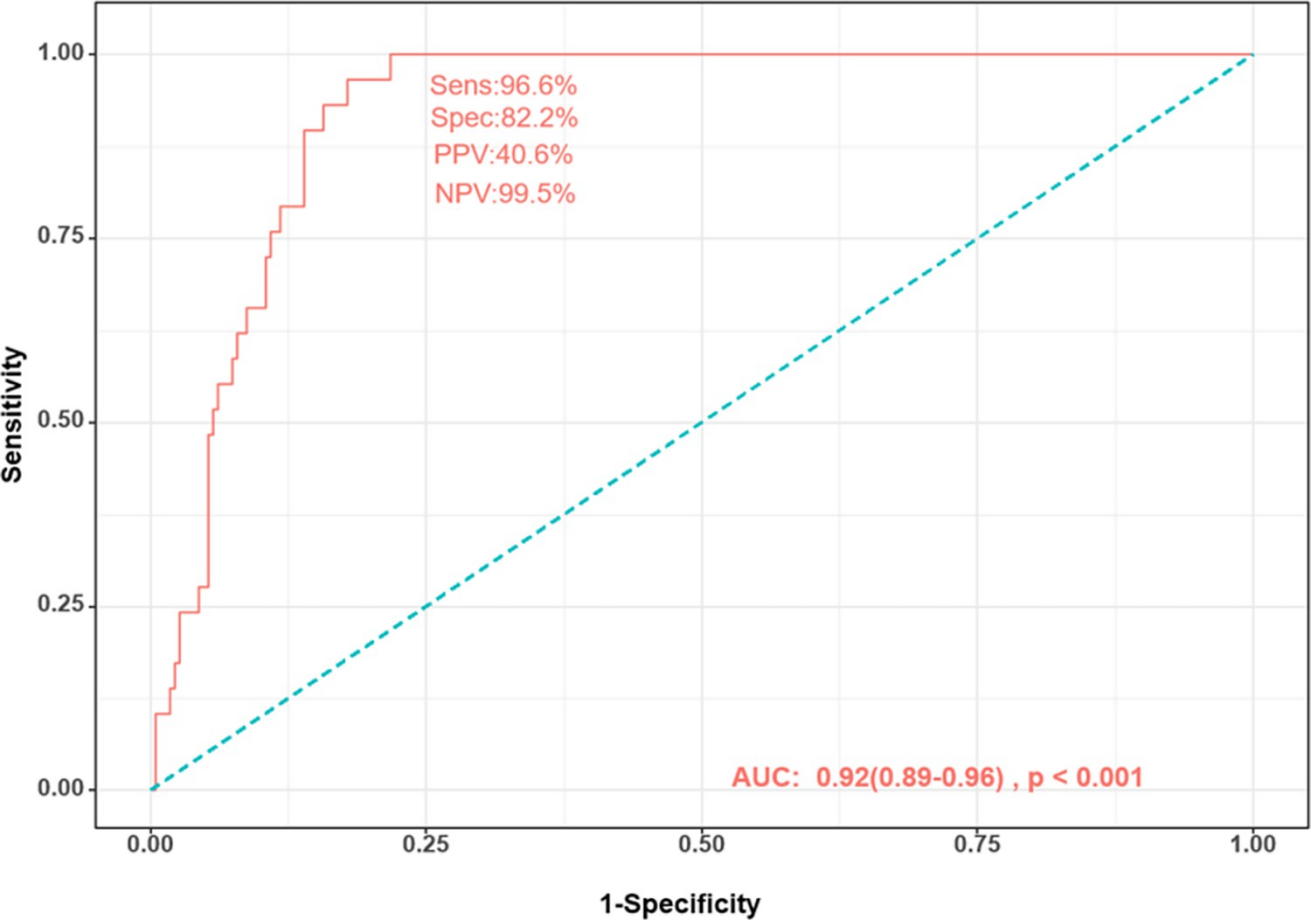
Receiver operating characteristic curve generated with 9 species for the diagnosis of GD: *Sutterella*, *Roseburia*, *Escherichia-shigella*, *Lachnospiraceae NK4A136 group*, *Parasutterella*, *Anaerostipes*, *Bilophila*, *f*.*Enterobacteriaceae*, *and Incertae Sedis*. GD, Graves’ disease; PPV, positive predictive value; NPV, negative predictive value.

We identified the top nine microbiota, with an LDA scored greater than 3.0, to serve as biomarkers for the diagnosis of GD, including *Sutterella*, *Roseburia*, *Escherichia-shigella*, *Lachnospiraceae NK4A136 group*, *Parasutterella*, *Anaerostipes*, *Bilophila*, *f*.*Enterobacteriaceae*, and *Incertae Sedis*. An ROC curve was generated to evaluate the diagnostic accuracy based on these 9 species, revealing an AUC of 0.92 (p < 0.001), sensitivity of 96.6%, specificity of 82.2%, positive predictive value of 40.6%, and negative predictive value of 99.5% (Fig 8). The variables importance to the classification model is shown in S1 Fig.

#### 4) Gut microbiota in smokers and non-smokers in GD

Among GD patients, two genera with more than 1% in relative abundance showed significant differences between smoker and non-smokers. Smokers exhibited a significantly higher relative abundance of *Faecalibacterium* (10.0 ± 3.6 versus 5.6 ± 3.9, p = 0.014), while *Dialister* displayed a lower abundance in smokers (0.0 ± 0.0 versus 2.2 ± 4.6, p = 0.014). Additionally, the genus *Anaerostipes* showed higher abundance in smokers among GD patients (0.2 ± 0.4 versus 0.8 ± 1.0, p = 0.16).

### Difference in gut microbiota in GD before and after treatment

**Fig 9:**
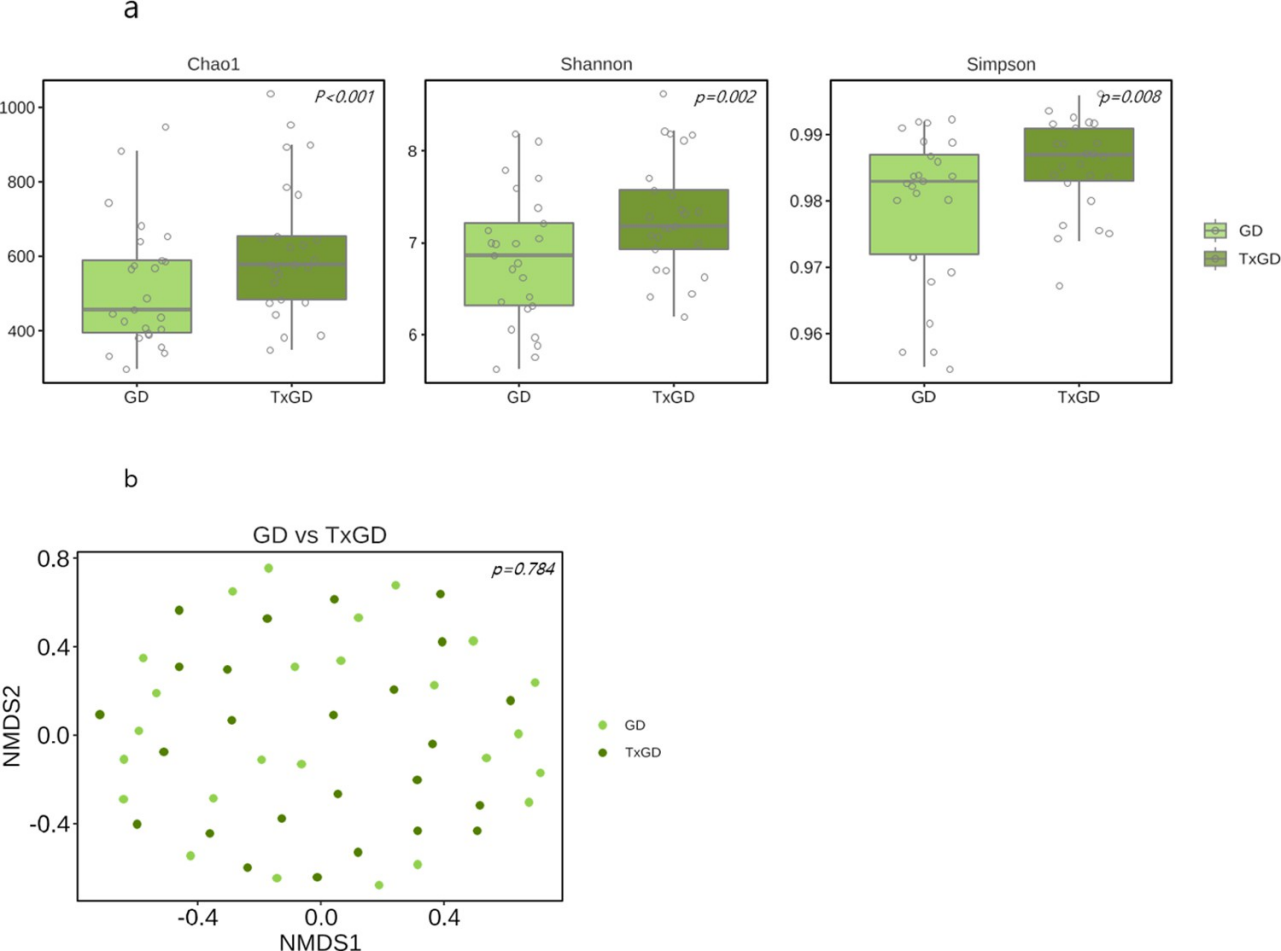
Difference in gut microbiota before and after anti-thyroid drug treatment (n = 25). (a) Alpha diversity between GD and TxGD. (b) Beta diversity between GD and TxGD. GD, Graves’ disease; TxGD, Graves’ disease patients after 6 months treatment with anti-thyroid drug; NMDS, non-metric multidimensional scaling.

Fig 9 shows the change in the alpha diversity in 25 GD patients 6 months after ATD treatment. Post-treatment, there is a significant increase in alpha diversity across all Chao1, Shannon, and Simpson indexes (p-value; 0.003, 0.002, 0.008 respectively). However, there is no a significant difference in the dissimilarity of beta diversity at the ASV level after treatment (p = 0.784) (Fig 9).

#### 1) Difference in gut microbiota at phylum level

**Fig 10:**
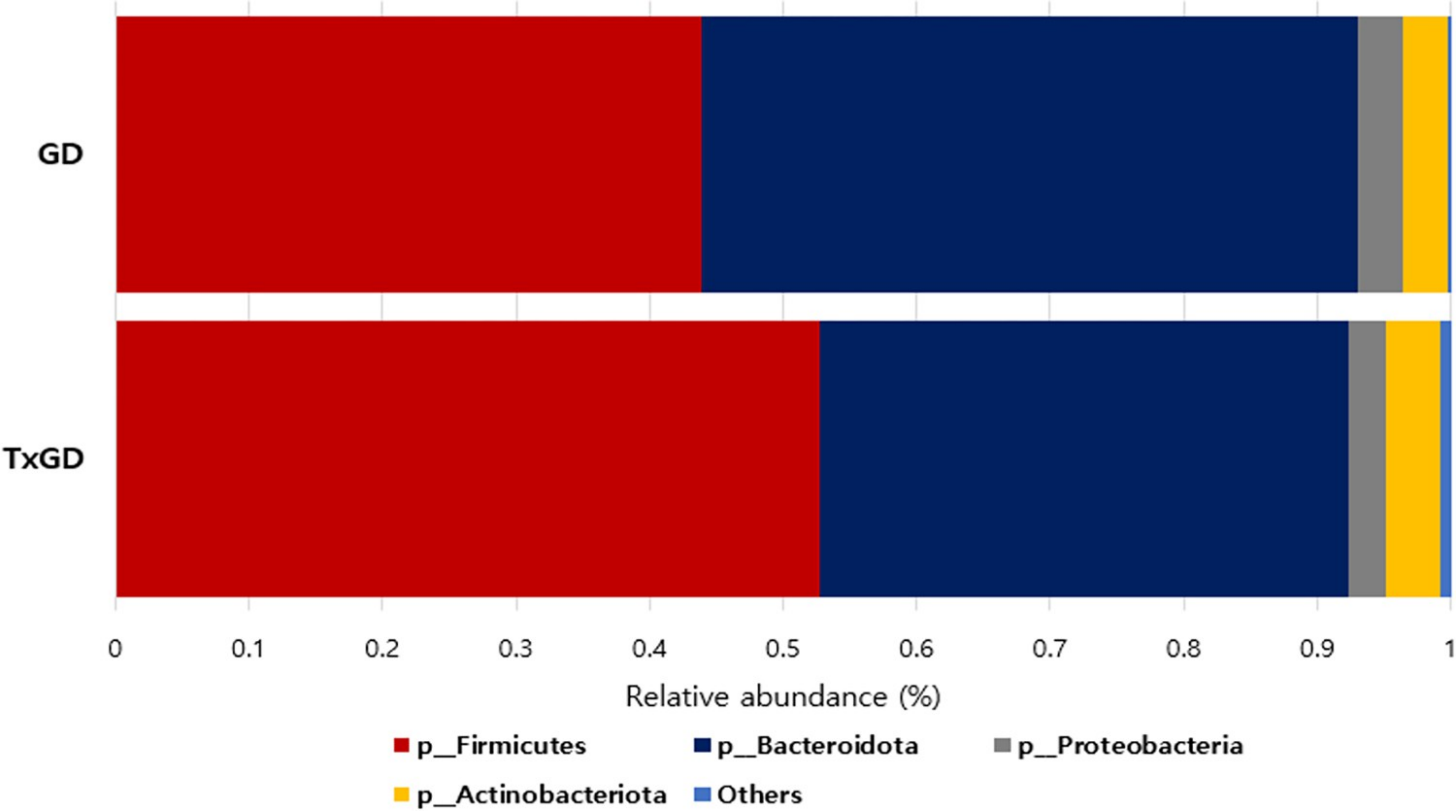
Difference in taxonomy composition in GD and TxGD at phylum level (n = 25). GD, Graves’ disease; TxGD, Graves’ disease patients after 6 months treatment with anti-thyroid drug.

**Fig 11:**
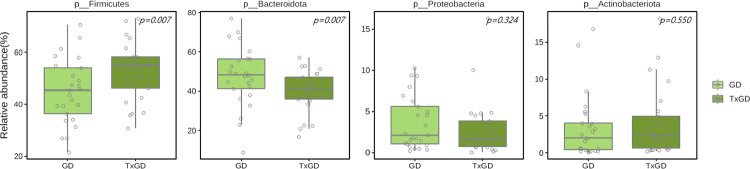
Differences in bacterial phyla between GD and TxGD (n = 25). GD, Graves’ disease; TxGD, Graves’ disease patients after 6 months treatment with anti-thyroid drug.

Fig 10 and S4 Table shows the alterations in composition in the phylum level following ATD treatment. Firmicutes increased significantly after treatment (p = 0.015), while Bacteroidota decreased in the post-treatment GD group (p = 0.014). At the phylum level, Proteobacteria and Actinobacteriota show no significant differences (p = 0.324, p = 0.550, respectively) (Fig 11). The relative abundance of Firmicutes/Bacteroidota increased from 1.2 ± 1.3 in pretreatment GD to 1.5 ± 0.9 in post-treatment GD group, although this trend was not statistically significant (p = 0.258).

#### 2) Difference in gut microbiota at genus level

**Fig 12:**
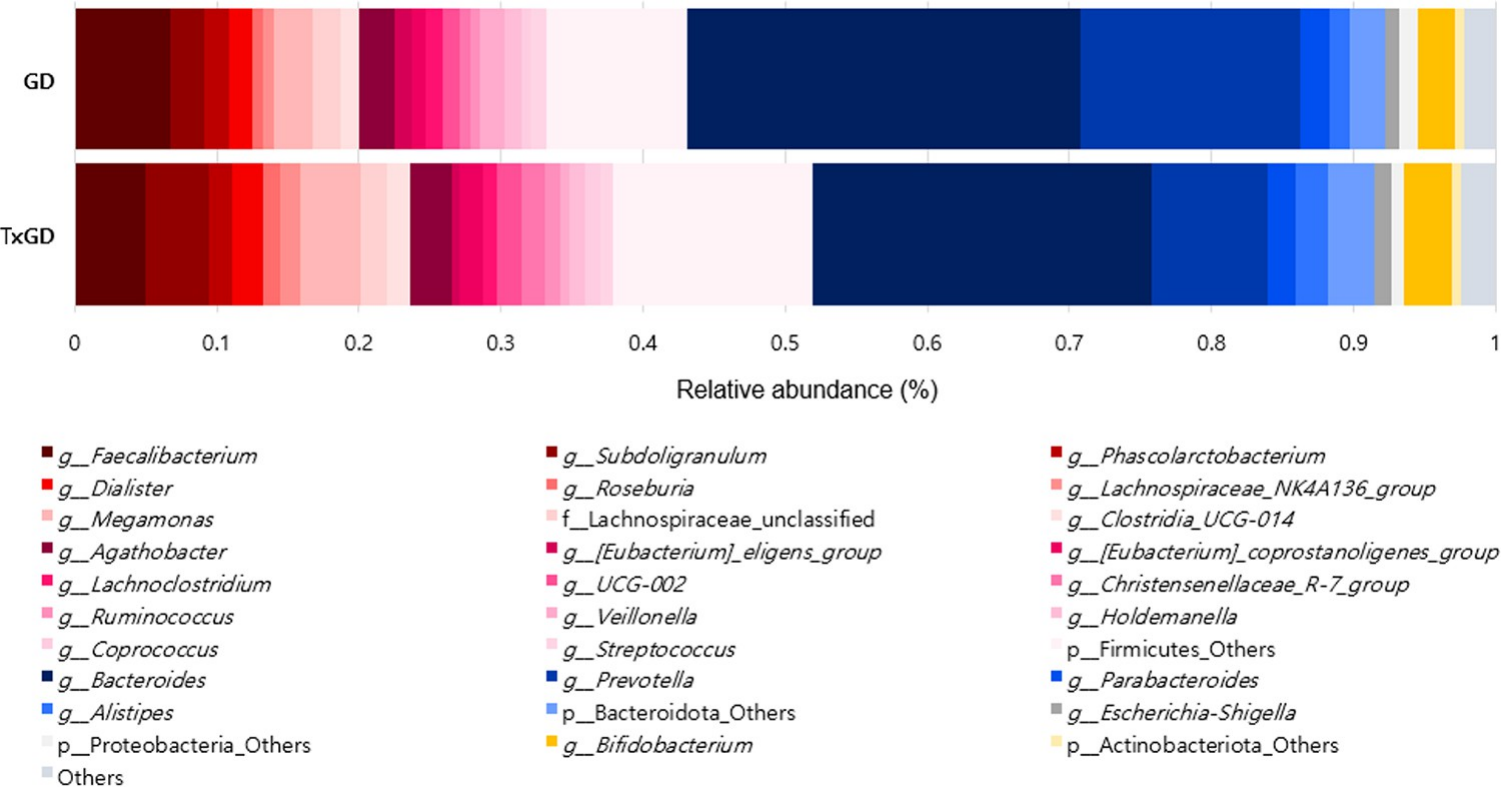
Difference in taxonomy composition in GD and TxGD at genus level (n = 25). GD, Graves’ disease; TxGD, Graves’ disease patients after 6 months treatment with anti-thyroid drug.

**Fig 13:**
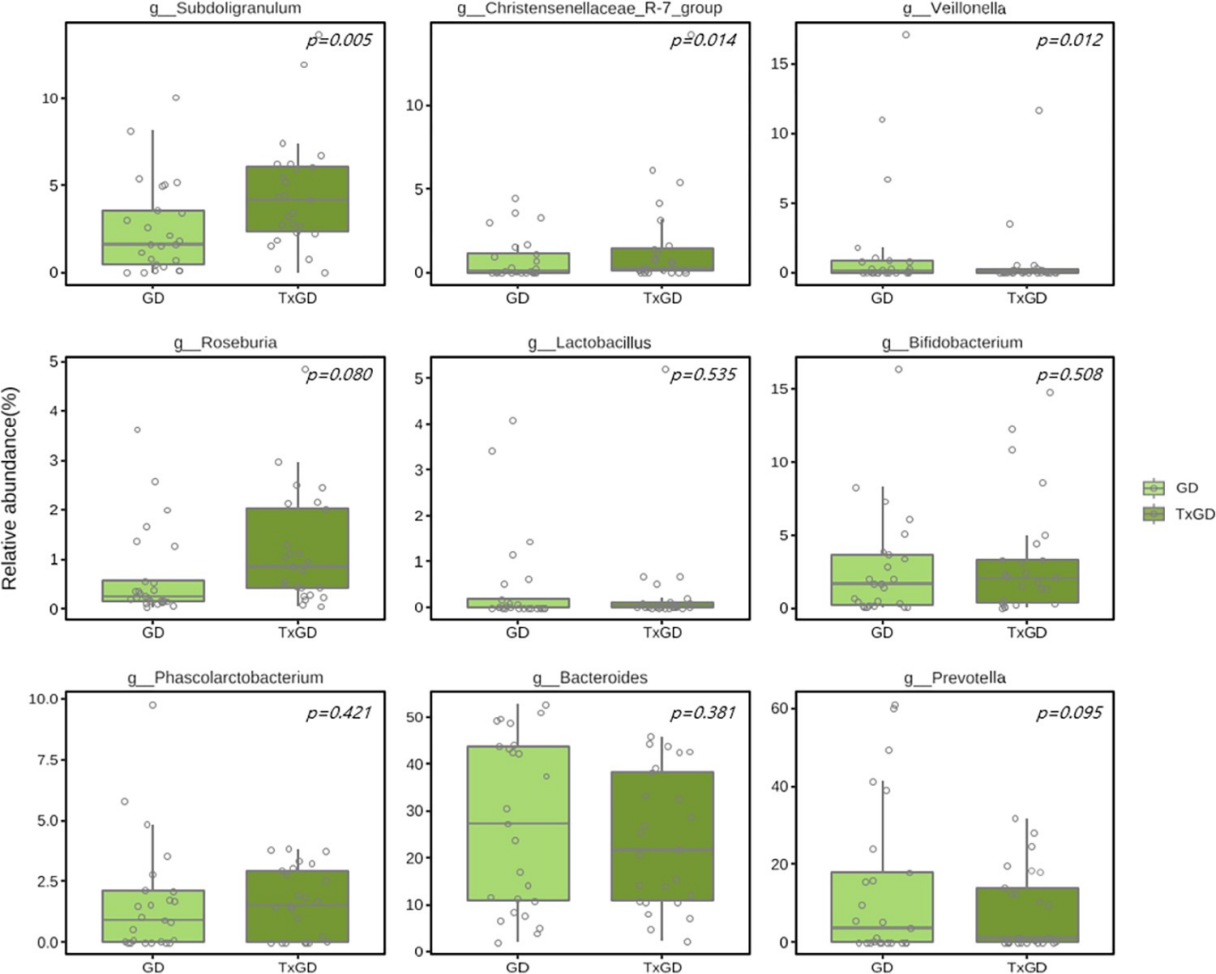
Differences in bacterial genus between GD and TxGD (n = 25). GD, Graves’ disease; TxGD, Graves’ disease patients after 6 months treatment with anti-thyroid drug.

**Fig 14:**
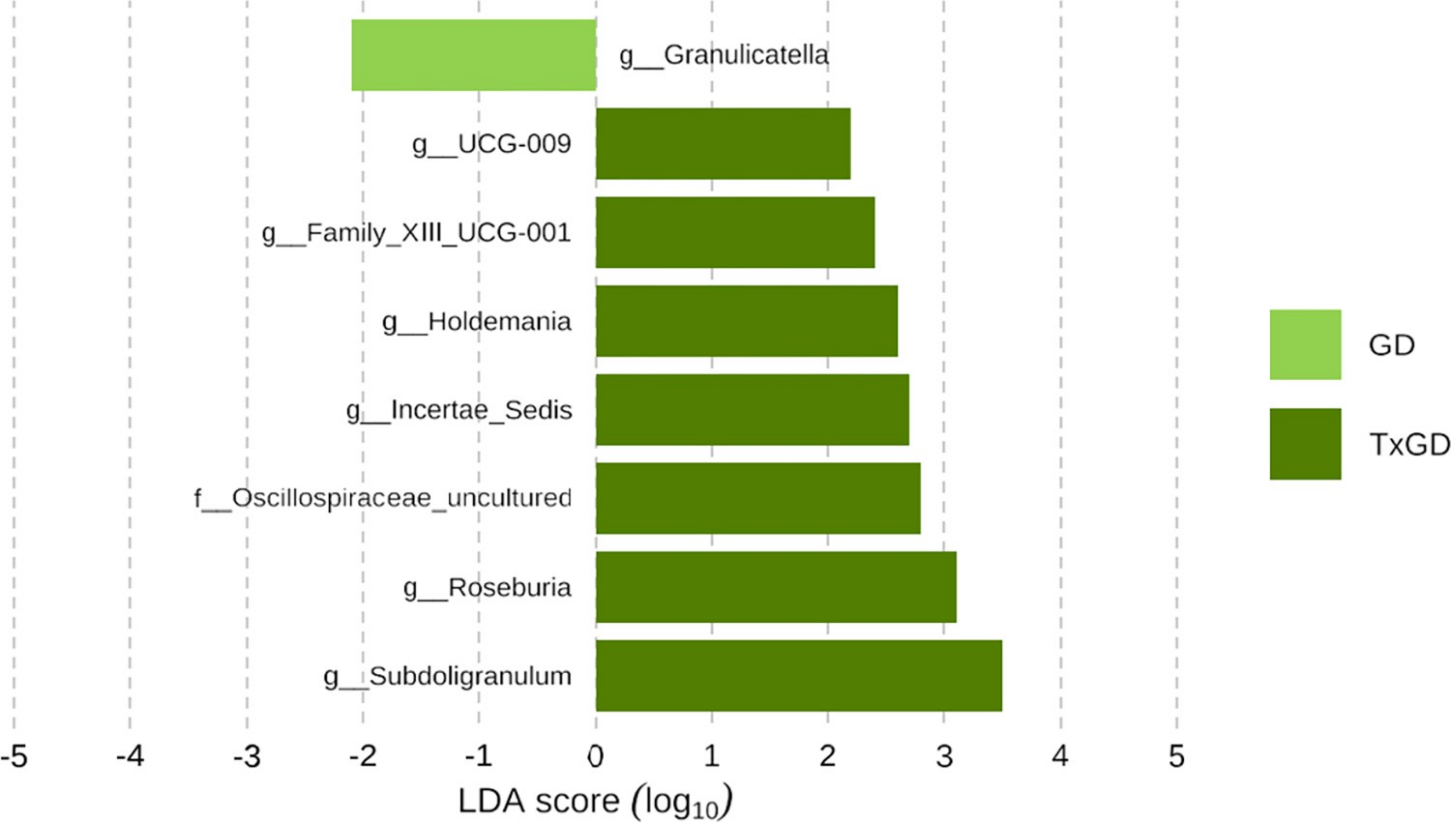
Distinguishment in bacterial genus between GD and TxGD by LEfSe algorithm (n = 25). GD, Graves’ disease; TxGD, Graves’ disease patients after 6 months treatment with anti-thyroid drug.

Fig 12 and S5 Table shows the alterations in composition at the genus level in the microbiota with relative abundance greater than 1%. The relative abundance of each genus before and after treatment is detailed in S5 Table. *Subdoligranulum* significantly increased after treatment (p = 0.010), while *Veillonella* and *Christensenellaceae R-7 group* both significantly decreased after treatment (p = 0.023, p = 0.029, respectively) (Fig 13). We also identified 2 genera that could significantly distinguish the two groups using the LEfSe algorithm (Linear discriminant analysis >3.0) (Fig 14).

### Difference in gut microbiota between and HC and GD after treatment

S2 Fig displays the differences in alpha diversity between 25 GD patients after ATD treatment and the HC group. Notably, there were no statistically significant differences between the two groups in alpha diversity, as indicated by the Chao1, Shannon, and Simpson indexes (p-values: 0.729, 0.064, and 0.071, respectively). The taxonomic composition in GD patients after ATD treatment and HC groups at the phylum and genus levels is presented in S3A and S3B Fig. Following treatment, there were no statistically significant differences between the two groups in phylum Firmicutes and Bacteroidota (p-values: 0.087 and 0.434, respectively) (S4 Fig).

### Results of correlation analysis

#### 1) Spearman correlation analysis at phylum level

At the phylum level, there was a positive association between Bacteroidota and SRS (correlation coefficient [CC] 0.59, p = 0.004), while Firmicutes exhibited a negative association with SRS (CC -0.73, p = 0.007).

#### 2) Spearman correlation analysis at genus level

**Fig 15:**
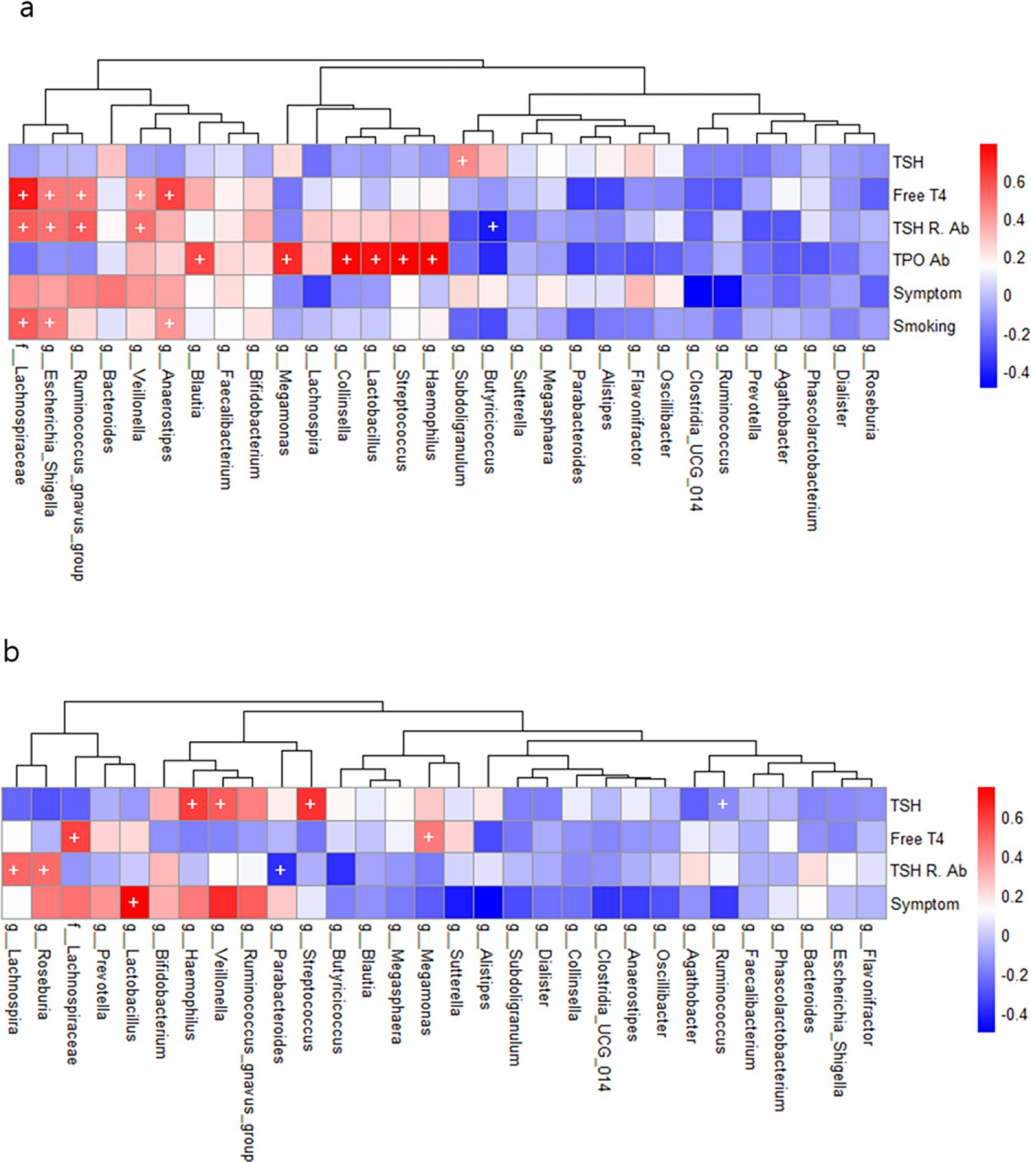
Heatmap of correlations analysis of gut microbiota. Symbols (+) refer to p-values < 0.05. (a) Correlations before treatment. (b) Correlations after treatment.

In Fig 15, the heatmap illustrates the correlation analysis of laboratory finding with microbiota at genus level before and after treatment. At baseline, several genera were associated with TSHR-Ab: *Veillonella* (CC 0.49, p = 0.007), *Escheria-shigella* (CC 0.50, p = 0.005), *Ruminococcus gnavus* (CC 0.55, p = 0.002), and *Butyricicoccus* (CC -0.42, p = 0.021). Moreover, *unclassified_f_Lachnospiraceae* (CC 0.71, p < 0.001), *Veillonella* (CC 0.40, p = 0.031), *Escheria-shigella* (CC 0.47, p = 0.011), *Ruminococcus gnavus* (CC 0.46, p = 0.011), and *Anaerostipes* (CC 0.61, p < 0.001) were associated with free T4 level. Some genera correlated with smoking pack years including *Escheria-shigella* (CC 0.45, p = 0.01), and *Anaerostipes* (CC 0.40, p = 0.03).

## Discussion

The present study investigated the gut microbiome in individuals newly diagnosed GD and observed shifts in microflora before and after treatment. The study unveiled a notable distinction in diversity between GD patients and healthy controls. Remarkably, post-treatment, the perturbed diversity in GD patients was restored.

The gut-associated lymphoid tissue harbors a diverse population of T-cells, including pro-inflammatory helper T (Th) cells and anti-inflammation regulatory T (Treg) cells. The intricate balance between these subsets is crucial for immune homeostasis. In the context of autoimmune diseases, the level of Th17 cells play a significant role [25, 26]. GD patients have been reported to have high levels of circulating Treg cell alongside an increased abundance of Th17 cells [27, 28]. Increased Th17 and impaired Treg responses are implicated in pathogenesis of GD. It has been reported that gut microbiome play a role in maintaining the dynamic balance between Th17 and Treg cells [29]. Numerous gut microorganisms have the capacity to produce short chain fatty acids (SCFAs) such as acetic acid, propionic acid, and butyric acid. These SCFAs have demonstrated regulatory effect on the generation of Th1 and Th17, while activating Treg cell activity [30–32]. By enhancing the integrity of the intestinal barrier function, SCFA may play a crucial role in shaping the development of GD.

*Roseburia*, known for its production of SCFAs such as acetic acid and butyric acid [33], was significantly decreased in GD patients, which is consistent with the previous research outcomes [34]. Furthermore, we observed an increase in *Roseburia* after ATD treatment, although this change did not reach statistical significant. As discussed earlier, the production of SCFAs has potential to induce Tregs cells and enhance their function [35]. For instance, in patients with ulcerative colitis, there was a discernible decrease in abundance of *Roseburia* bacteria, which is associated with concurrent reduction in concentrations of SCFAs [36, 37].

Indeed, the beneficial effect of SCFAs are not universal across all types. Unlike *Roseburia*, *Bacteroides* has the capacity to produce various SCFAs other than butyric acid, including succinic acid, propionic acid, and acetic acid. Importantly, unlike butyrate, these SCFAs do not induce mucin synthesis. The consequence of this is reduction in intestinal tight junctions as well as increased permeability of the gut mucosa [38, 39]. In GD patients, there was increase in the abundance of *Bacteroides*, which exhibited a subsequent decrease after treatment. As suggested, the elevated presence of *Bacteroides* may contribute to the release of pro-inflammatory factors beyond the intestine, potentially leading to immune dysfunction [13]. Similar Increase in *Bacteroides* have been documented in other autoimmune conditions, including rheumatoid arthritis and type 1 diabetes [38, 40]. *Bacteroides* may play an auxiliary role in the pathophysiology of GD. Notably, two studies have reported a significantly higher abundance of *Bacteroides* in GD patients compared to the healthy controls [13, 41]. However, conflicting findings have been reported in other studies [42, 43]. Therefore, further studies are needed to clarify the specific impact of *Bacteroides* on the thyroid.

The level of *Subdoligranulum* was found to be lower in GD patients, exhibiting a significant increase after treatment. Additionally, a notable negative correlation was observed between TSHR-Ab and free T4 level, implying its potential influence on the pathogenesis of GD. Likewise, several diseases—such as IBD—were associated with a lower abundance of butyrate producers like *Subdoligranulum* [44, 45].

Our data revealed an non-significant change in the level of *Lactobacillus* and *Bifidobacterium*, which is consistent with the findings in other studies [7, 13, 41]. Lactate-producing beacteria, *Lactobacillus* and *Bifidobacterium*, are typically considered to be beneficial microbiota in various contexts. Probiotics, which generally contain *Lactobacillus* and *Bifidobacterium* genera, has demonstrated positive effect, including a reduction in colorectal cancer and therapeutic potential against obesity [10, 46]. Certainly, while Lactobacillus and Bifidobacterium are generally considered beneficial, there are exceptions in specific diseases. For instance, increases in the abundance of *Lactobacillus* have been reported in autoimmune hepatitis [47] and Crohn’s disease [48]. Delcenseri et al. [49] suggested that probiotics may induce inflammation through the production of Th1 cells, leading to a negative influence on autoimmune diseases mediated by Th1 cells. As previously mentioned, our data indicated a decrease in *Lactobacillus* after ATD treatment, which is consistent with previous works [41, 50]. Furthermore, several studies have shown a positive correlation with *Lactobacillus* and thyroid antibody of TSHR-Ab and TPO Ab, suggesting its detrimental effect on GD pathogenesis [13, 41, 51].

By contrast, we speculate that *Bifidobacterium* may exert a probiotic effect on GD that differs from *Lactobacillus*. Our data showed an insignificant increase in *Bifidobacterium* after ATD treatment. *Bifidobacterium* supplementation has been recognized for its ability to inhibit the over-activation of Th17 [52].

At the phylum level, Firmicutes and Bacteroidota emerged as the dominant microbiota in individuals newly diagnosed with GD. The proportion of Firmicutes was lower in the GD group compared to HC, while the proportion of Bacteroidota was higher in the GD group than in the HC.

Notably, the contrasting finding in patients with Hashimoto’s thyroiditis or thyroid cancer, where Firmicutes were more abundant and Bacteroidota were less abundant [9, 53], suggesting a potential difference in pathogenesis of these diseases. The ratio of Firmicutes to Bacteroidetes has been proposed in several previous studies as indicator of the health status [54–56]. As demonstrated in our findings, the reduced ratio of Firmicutes to Bacteroidota was consistent with the results from other studies examining both GD and Graves’ ophthalmopathy (GO) patients [7, 13, 57, 58]. Interestingly, these imbalances were observed to be restored after ATD treatment.

Smoking is recognized for its pro-inflammatory effect and is a well-known as aggravating factor of GD and GO [59]. However, the precise mechanism by which smoking enhance immune pathways like Th2 responses leading to Graves’ disease, remain unclear [60]. In our study, we observed a positive association between *Anaerostipes* and smoking pack years, indicating that *Anaerostipes* was more abundant in smokers among GD patients. Consistently, *Anaerostipes* was linked with tobacco usage in IBD patients [61]. Additionally, our date revealed a significant association between *Anaerostipes* and TSHR-Ab. Quadbeck et al. [62] determined that smokers had a higher level of TSHR-Ab than nonsmokers. Based on these findings, we speculate that smoking might induce the pathogenic effect of *Anaerostipes* on autoimmunity, potentially aggravating both GD and GO.

In our study, we observed that alpha diversity was lower in GD patients compared to HC group, and this diversity was recovered after GD treatment. This aligns with findings from several prior studies that have consistently reported reduced richness of gut microbiome in GD patients compared to healthy controls [7, 13, 28, 41, 58, 63]. By contrast, there is one study [57] reporting a slight non-statistically significant increase in microbial diversity and richness compared to HC. However, considering the observed increase in alpha diversity after treatment and overall improvement in the microflora, it is reasonable to speculate that alpha diversity decreases in GD. Similar decreases in alpha diversity have also been observed in IBD [64] and autoimmune hepatitis patients [47].

We found a negative association of the phylum Firmicutes and a positive association of Bacteroidota with the symptoms of GD. Interestingly, Firmicutes and Bacteroidota did not show any correlation with free T4 level or T3 level, hormones typically associated with symptoms like nervousness, sweating, tremor, and hyperactivity. To our knowledge, this is the first research to report an association of GD symptoms with intestinal flora.

Typically, the diagnosis of GD involves the measurement of TSHR-Ab and radionuclide scanning of the thyroid is typically. However, it takes several days to receive the results of these assessments, particularly at primary physician clinics. Moreover, radionuclide thyroid scans can only be performed in a limited number of equipped hospitals and are not suitable for pregnant women. The advancement in microbiome research suggest that the non-invasive sampling of microbiome could offer an alternative for the convenient diagnosis of GD. The diagnostic efficacy of GD using stool samples showed a 96.6% sensitivity and an 82.2% specificity with an AUC value of 0.923. These values closely align with finding of Jiang et al. [13], which showed an AUC value of 0.811 using the same top nine species predicted by LEfSe analysis. However, it is essential to recognize that while this approach holds promise, it may not completely replace established methods. Microbiome profiling, based on 16S rRNA gene sequencing, also requires several days for completion, is influenced by various factors, and may encounter challenges related to data interpretation and standardization. Further development and refinement of microbiome profiling are essential for the potential replacement current assessment tools. Furthermore, it is important to note that while our classifier demonstrates strong discriminatory power between Graves’ disease patients and healthy controls, its true clinical utility lies in its ability to differentiate between Graves’ disease and other symptomatic thyroid disorders. Further validation of this utility is warranted, especially in cohorts exhibiting symptoms of hyperthyroidism.

Our study has several limitations. First, the composition and activities of the gut microbiome could be affected by various factors such as age, diet, race, and drugs [65]. Therefore, our results may be specific to the Korean population. Secondly, the GD patients in our study were not treated with the same medicine; as mentioned, methimazole, carbimazole, and propylthiouracil were used for the treatment of GD. Sun et al. [50] showed that ATD itself caused changes in the gut microbiome structure. Unfortunately, this study did not account for the specific interference of each ATD treatment. Thirdly, we chose to employ 16S rDNA sequencing in our research. This choice is significant given the increasing popularity of shotgun metagenomic sequencing for gut microbiota studies. While shotgun metagenomics sequencing provides a powerful tool for identifying microorganisms at the species or even strain level, it’s essential to recognize that 16S rDNA sequencing remains a widely used method in the field of gut microbiome research, even in studies on Graves’ disease [3, 7, 13, 34, 42, 43, 57, 66]. The utilization of 16S rDNA sequencing enhances comparability and contributes to a more comprehensive understanding of the gut microbiota. Fourth, the open database we utilized for this study did not provide access to the laboratory findings or clinical parameters of each healthy control. Therefore, we were unable to include the thyroid-related indicators for the healthy controls in our research. Lastly, the small sample size is a limitation. However, a notable aspect of our work is the comparison made before and after anti-thyroid drug treatment, which is a relatively rare perspective in the field. Despite the sample size limitation, we believe our research provide valuable insights into microbiome analysis in patients with Graves’ disease.

## Conclusion

The pathogenesis of GD has not been fully elucidated. Our research not only pointed out the distinctive features of gut microbiota in GD patients but also demonstrated their alternation and subsequent restoration following ATD treatment. This supports the hypothesis that the gut microbiome may indeed play a role in the pathogenesis of GD.

## Supporting information

S1 Fig The variables importance score to the classification model. (TIF)

S2 Fig Alpha diversity between Tx GD (n = 25) and HC (n = 130). TxGD, Graves’ disease patients after 6 months treatment with anti-thyroid drug; HC, healthy control.
(TIF)

S3 Fig Taxonomy composition in Tx GD (n = 25) and HC (n = 130) at phylum level. (a) Taxonomy composition in GD and TxGD at phylum level. (b) Taxonomy composition in GD and TxGD at genus level. TxGD, Graves’ disease patients after 6 months treatment with anti-thyroid drug; HC, healthy control.
(TIF)

S4 Fig Differences in bacterial phyla between Tx GD (n = 25) and HC (n = 130). TxGD, Graves’ disease patients after 6 months treatment with anti-thyroid drug; HC, healthy control.
(TIF)

S1 Table Hyperthyroid symptom scale. (DOCX)

S2 Table The baseline relative abundance of each phylum in healthy controls and Graves’ disease patients. (DOCX)

S3 Table The baseline relative abundance of each genus in healthy controls and Graves’ disease patients. (DOCX)

S4 Table The relative abundance of each phylum in Graves’ disease patients before and after treatment. (DOCX)

S5 Table The baseline relative abundance of each genus in Graves’ disease patients before and after treatment. (DOCX)
