# Supplementary figures and images for: Gut microbiome in the Graves’ disease: Comparison before and after anti-thyroid drug treatment
Source: PLoS One. 2024 May 31;19(5):e0300678. doi: 10.1371/journal.pone.0300678 (PMC11142679; doi:10.1371/journal.pone.0300678)

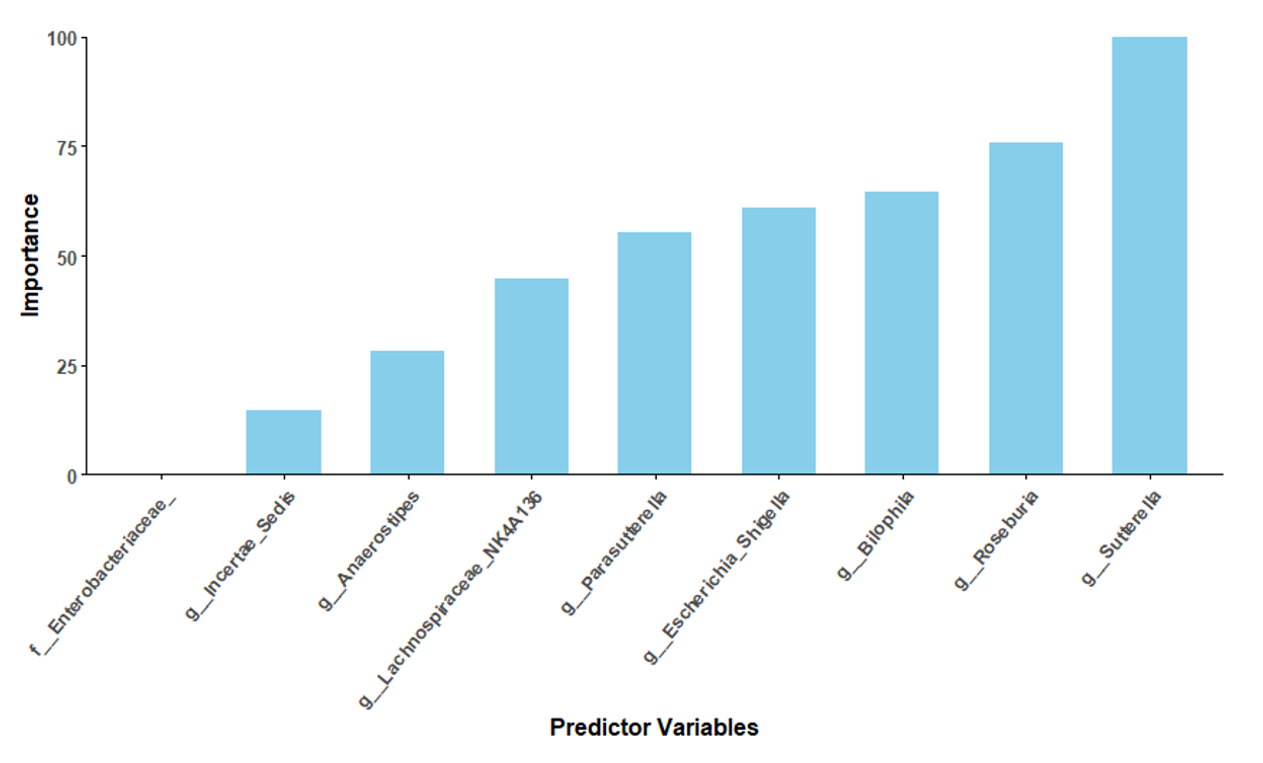

Supplement: S1 Fig — (TIF) [file pone.0300678.s001.tif]

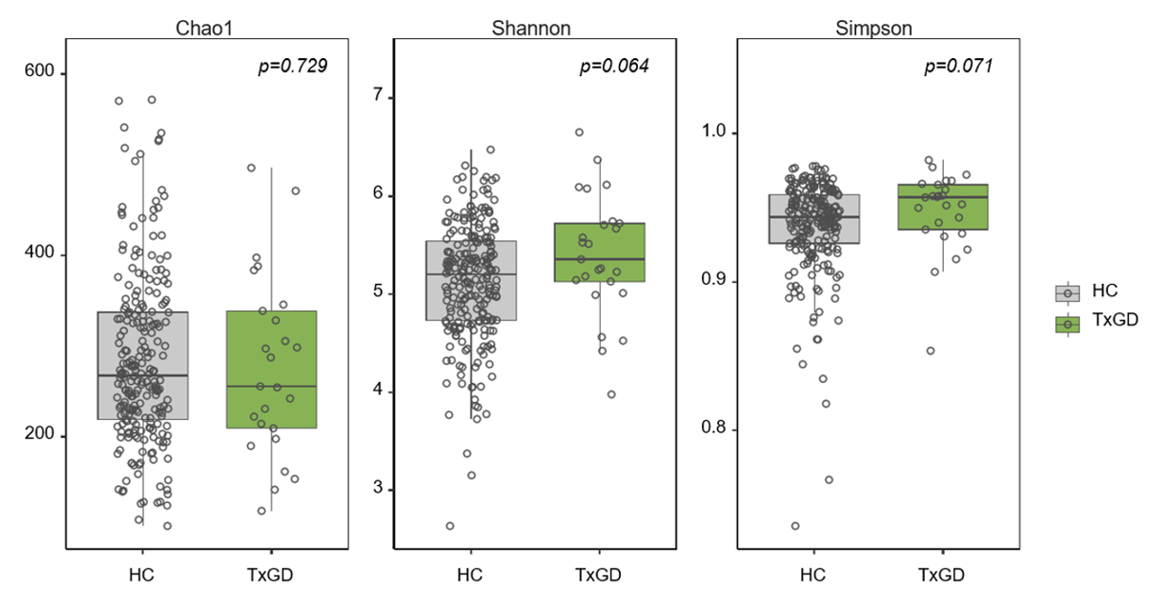

Supplement: S2 Fig — TxGD, Graves’ disease patients after 6 months treatment with anti-thyroid drug; HC, healthy control. (TIF) [file pone.0300678.s002.tif]

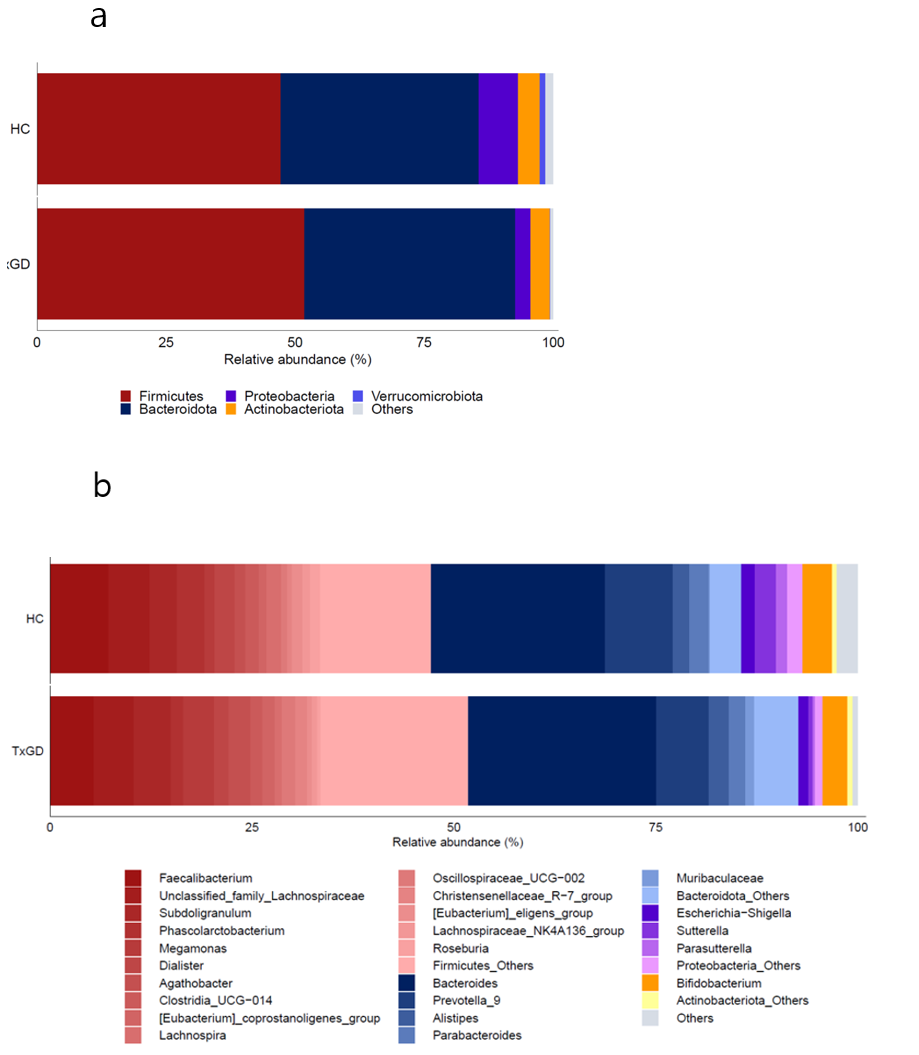

Supplement: S3 Fig — (a) Taxonomy composition in GD and TxGD at phylum level. (b) Taxonomy composition in GD and TxGD at genus level. TxGD, Graves’ disease patients after 6 months treatment with anti-thyroid drug; HC, healthy control. (TIF) [file pone.0300678.s003.tif]

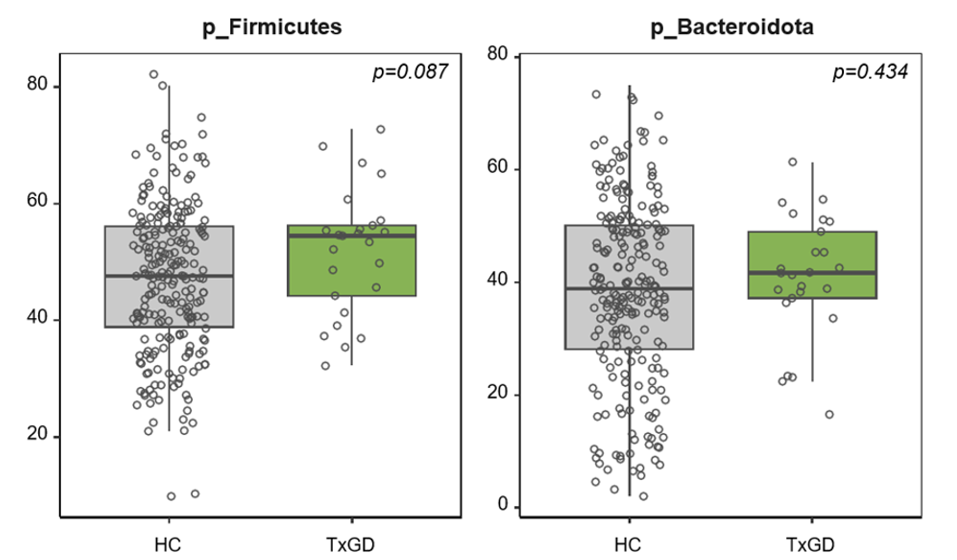

Supplement: S4 Fig — TxGD, Graves’ disease patients after 6 months treatment with anti-thyroid drug; HC, healthy control. (TIF) [file pone.0300678.s004.tif]
