# Supplementary material for: Gut microbiome in the Graves’ disease: Comparison before and after anti-thyroid drug treatment
Source: PLoS One. 2024 May 31;19(5):e0300678. doi: 10.1371/journal.pone.0300678 (PMC11142679; doi:10.1371/journal.pone.0300678)
Supplement: S1 Table — (DOCX) [file pone.0300678.s005.docx]

S1 table

Hyperthyroid Symptom Scale

| **Characteristic** | **Score** |
| --- | --- |
| **Nervousness**  O. Absent  1. Anxious only with stress  2. Occasionally anxious at rest  3. Often anxious, difficulty working or concentrating  4. States freely that feels "very nervous most of the time" |  |
| **Sweating**  O. Only with activity  1. At rest but only in warm temperatures  2. At rest in temperate climates, mainly involving the hands and intertriginous zones  3. At rest involving many areas  4. Profusely diaphoretic almost constantly |  |
| **Heat tolerance**  O. Normal temperature tolerance  1. Periods of feeling warmer than those in the same room  2. Significant difficulty with heat, requiring air conditioner constantly in the summer time  3. Excessive difficulty with heat even in temperate climates  4. Extreme difficulty with heat, does feel comfortable even in cold weather as evidenced by lack of need for warm clothing or bed covers |  |
| **Hyperactivity**  0. Normal activity level  1. Increased activity level, increased productivity  2. Increased productivity; decreased sleep time  3. Performs some purposeless activity  4. Frequent episodes of purposeless activity; unable to sit still during examination |  |
| **Tremor: Examination of outstretched hands**  0. Absent  1. Barely perceptible  2. Tremor demonstrated readily on examination  3. Marked tremor but able to perform fine motor skills  4. Hands shake excessively, difficulty performing fine motor skills |  |
| **Weakness**  0. Normal strength  1. Subjective weakness but with normal exercise tolerance  2. Decreased exercise tolerance to near maximal activity  3. Decreased tolerance to stair climbing or arising from chair  4. Extreme weakness such that patient can barely lift objects or walk up stairs |  |
| **Hyperdynamic precordium**  0. Normal precordium activity and apical impulse  1. Tachycardia, 90 beats per minute with normal apical impulse  2. Tachycardia, 90 beats per minute with apical impulse  3. Tachycardia, 110 beats per minute with increased apical impulse  4. Tachycardia, 110 per minute, apical impulse and carotid upstroke increased, systolic outflow murmur |  |
| **Diarrhea**  0. 1 bowel movement (BM) per day; formed stool  1. 2-4 BMs per day  2. 1-4 loose stools per day  3. 4 formed BMs per day  4. 4 stools per day |  |
| **Appetite**  1. Appetite normal, no weight loss  2. Appetite normal, weight loss  3. Appetite increased, no weight loss  4. Appetite increased, weight loss  5. Appetite decreased, weight loss |  |
| **Assessment of daily function (degree of incapacitation)**  0. Normal (none)  1. Minimal impairment (10%)  2. Mild impairment (30%)  3. Moderate impairment (60%)  4. Severe impairment (90%) |  |
