# Supplementary material for: Gut microbiome in the Graves’ disease: Comparison before and after anti-thyroid drug treatment
Source: PLoS One. 2024 May 31;19(5):e0300678. doi: 10.1371/journal.pone.0300678 (PMC11142679; doi:10.1371/journal.pone.0300678)
Supplement: S2 Table — (DOCX) [file pone.0300678.s006.docx]

S2 table

The baseline relative abundance of each phylum in healthy controls and Graves’ disease patients

| Phylum | Healthy control | Graves’ disease |
| --- | --- | --- |
|  | (N=230) | (N=29) |
| p;Firmicutes | 47.33 ± 13.02 | 43.83 ± 13.41 |
| p;Proteobacteria | 7.99 ± 5.30 | 3.47 ± 3.11 |
| p;Bacteroidota | 37.17 ± 16.11 | 49.14 ± 15.89 |
| p;Actinobacteriota | 4.65 ± 7.14 | 3.27 ± 4.29 |
| p;Patescibacteria | 0.02 ± 0.04 | 0.02 ± 0.07 |
| d;Bacteria;_ | 0.02 ± 0.02 | 0.01 ± 0.02 |
| p;Verrucomicrobiota | 1.20 ± 3.31 | 0.01 ± 0.03 |
| p;Fusobacteriota | 0.34 ± 1.67 | 0.04 ± 0.10 |
| p;Desulfobacterota | 0.97 ± 1.81 | 0.18 ± 0.25 |
| p;Cyanobacteria | 0.21 ± 1.68 | 0.00 ± 0.02 |
| p;Campilobacterota | 0.01 ± 0.12 | 0.01 ± 0.02 |
| p;Synergistota | 0.09 ± 0.78 | 0.00 ± 0.00 |
| p;Spirochaetota | 0.00 ± 0.02 | 0.00 ± 0.00 |
| p;Elusimicrobiota | 0.00 ± 0.00 | 0.00 ± 0.01 |
| p;Deferribacterota | 0.00 ± 0.00 | 0.00 ± 0.00 |
| p;Acidobacteriota | 0.00 ± 0.00 | 0.00 ± 0.00 |
| p;Chloroflexi | 0.00 ± 0.00 | 0.00 ± 0.00 |
| Unassigned | 0.00 ± 0.00 | 0.00 ± 0.00 |

p; phylum

Data are expressed as mean ± standard deviation
