# Supplementary material for: Gut microbiome in the Graves’ disease: Comparison before and after anti-thyroid drug treatment
Source: PLoS One. 2024 May 31;19(5):e0300678. doi: 10.1371/journal.pone.0300678 (PMC11142679; doi:10.1371/journal.pone.0300678)
Supplement: S4 Table — (DOCX) [file pone.0300678.s008.docx]

S4 table

The relative abundance of each phylum in Graves’ disease patients before and after treatment

| Phylum | Before treatment | After treatment |
| --- | --- | --- |
|  | (N=25) | (N=25) |
| p;Firmicutes | 44.50 ± 13.20 | 52.75 ± 11.14 |
| p;Proteobacteria | 3.41 ± 3.13 | 2.92 ± 3.56 |
| p;Bacteroidota | 48.50 ± 16.33 | 39.53 ± 10.48 |
| p;Actinobacteriota | 3.30 ± 4.40 | 4.05 ± 4.58 |
| p;Patescibacteria | 0.02 ± 0.07 | 0.02 ± 0.04 |
| d;Bacteria;_ | 0.01 ± 0.02 | 0.01 ± 0.02 |
| p;Verrucomicrobiota | 0.01 ± 0.03 | 0.10 ± 0.33 |
| p;Fusobacteriota | 0.03 ± 0.08 | 0.23 ± 0.95 |
| p;Desulfobacterota | 0.21 ± 0.26 | 0.36 ± 0.36 |
| p;Cyanobacteria | 0.00 ± 0.02 | 0.00 ± 0.00 |
| p;Campilobacterota | 0.00 ± 0.02 | 0.00 ± 0.01 |
| p;Synergistota | 0.00 ± 0.0 | 0.00 ± 0.00 |
| p;Spirochaetota | 0.00 ± 0.00 | 0.00 ± 0.01 |
| p;Elusimicrobiota | 0.01 ± 0.01 | 0.02 ± 0.08 |
| p;Deferribacterota | 0.00 ± 0.00 | 0.00 ± 0.00 |
| p;Acidobacteriota | 0.00 ± 0.00 | 0.00 ± 0.00 |
| p;Chloroflexi | 0.00 ± 0.00 | 0.00 ± 0.00 |
| Unassigned | 0.00 ± 0.00 | 0.00 ± 0.00 |

p; phylum

Data are expressed as mean ± standard deviation
