# Supplementary material for: Gut microbiome in the Graves’ disease: Comparison before and after anti-thyroid drug treatment
Source: PLoS One. 2024 May 31;19(5):e0300678. doi: 10.1371/journal.pone.0300678 (PMC11142679; doi:10.1371/journal.pone.0300678)
Supplement: S5 Table — (DOCX) [file pone.0300678.s009.docx]

S5 table

The baseline relative abundance of each genus in Graves’ disease patients before and after treatment

| Genus | Before treatment | After treatment |
| --- | --- | --- |
|  | (N=25) | (N=25) |
| d;Bacteria_p;Firmicutes_c;Bacilli_o;Erysipelotrichales_f;Erysipelotrichaceae_g;Faecalitalea | 0.01 ± 0.03 | 0.01 ± 0.02 |
| d;Bacteria_p;Proteobacteria_c;Gammaproteobacteria_o;Pasteurellales_f;Pasteurellaceae_g;Haemophilus | 0.82 ± 1.98 | 0.67 ± 3.02 |
| d;Bacteria_p;Firmicutes_c;Clostridia_o;Peptococcales_f;Peptococcaceae;_ | 0.01 ± 0.02 | 0.01 ± 0.02 |
| d;Bacteria_p;Firmicutes_c;Clostridia;_ | 0.07 ± 0.10 | 0.08 ± 0.08 |
| d;Bacteria_p;Proteobacteria_c;Gammaproteobacteria_o;Enterobacterales_f;Enterobacteriaceae;_ | 0.41 ± 1.20 | 0.04 ± 0.12 |
| d;Bacteria_p;Bacteroidota_c;Bacteroidia_o;Bacteroidales_f;Rikenellaceae_g;Alistipes | 1.62 ± 2.43 | 2.23 ± 2.17 |
| d;Bacteria_p;Proteobacteria_c;Gammaproteobacteria_o;Burkholderiales_f;Sutterellaceae_g;Sutterella | 0.91 ± 1.25 | 0.53 ± 0.68 |
| d;Bacteria_p;Firmicutes_c;Clostridia_o;Lachnospirales_f;Lachnospiraceae_g;Lachnospiraceae_NC2004_group | 0.02 ± 0.03 | 0.03 ± 0.03 |
| d;Bacteria_p;Bacteroidota_c;Bacteroidia_o;Bacteroidales_f;Prevotellaceae_g;Paraprevotella | 0.69 ± 1.02 | 0.51 ± 0.59 |
| d;Bacteria_p;Firmicutes_c;Clostridia_o;Oscillospirales_f;Ruminococcaceae_g;Subdoligranulum | 2.58 ± 2.65 | 4.49 ± 3.26 |
| d;Bacteria_p;Firmicutes_c;Clostridia_o;Lachnospirales_f;Lachnospiraceae_g;Blautia | 0.43 ± 0.39 | 0.56 ± 0.57 |
| d;Bacteria_p;Actinobacteriota_c;Coriobacteriia_o;Coriobacteriales_f;Eggerthellaceae_g;Gordonibacter | 0.00 ± 0.00 | 0.00 ± 0.00 |
| d;Bacteria_p;Firmicutes_c;Clostridia_o;Lachnospirales_f;Lachnospiraceae;_ | 1.97 ± 2.39 | 1.90 ± 1.24 |
| d;Bacteria_p;Bacteroidota_c;Bacteroidia_o;Bacteroidales_f;Barnesiellaceae_g;Barnesiella | 0.28 ± 0.46 | 0.24 ± 0.28 |
| d;Bacteria_p;Bacteroidota_c;Bacteroidia_o;Bacteroidales_f;Prevotellaceae_g;Prevotellaceae_Ga6A1_group | 0.00 ± 0.00 | 0.00 ± 0.00 |
| d;Bacteria_p;Firmicutes_c;Clostridia_o;Peptococcales_f;Peptococcaceae_g;uncultured | 0.01 ± 0.03 | 0.03 ± 0.06 |
| d;Bacteria_p;Firmicutes_c;Clostridia_o;Lachnospirales_f;Lachnospiraceae_g;Lachnospiraceae_UCG_010 | 0.13 ± 0.14 | 0.19 ± 0.18 |
| d;Bacteria_p;Bacteroidota_c;Bacteroidia_o;Bacteroidales_f;Bacteroidaceae_g;Bacteroides | 27.48 ± 18.23 | 23.86 ± 14.39 |
| d;Bacteria_p;Firmicutes_c;Clostridia_o;Oscillospirales_f;Ruminococcaceae_g;Pygmaiobacter | 0.00 ± 0.00 | 0.00 ± 0.00 |
| d;Bacteria_p;Firmicutes_c;Clostridia_o;Oscillospirales_f;Ruminococcaceae_g;UBA1819 | 0.03 ± 0.06 | 0.11 ± 0.20 |
| d;Bacteria_p;Firmicutes_c;Clostridia_o;Lachnospirales_f;Lachnospiraceae_g;Lachnospira | 0.79 ± 0.70 | 0.81 ± 0.93 |
| d;Bacteria_p;Proteobacteria_c;Gammaproteobacteria_o;Burkholderiales_f;Sutterellaceae_g;Parasutterella | 0.13 ± 0.27 | 0.31 ± 0.80 |
| d;Bacteria_p;Firmicutes_c;Negativicutes_o;Veillonellales_Selenomonadales_f;Selenomonadaceae_g;Megamonas | 2.96 ± 6.64 | 4.24 ± 9.42 |
| d;Bacteria_p;Firmicutes_c;Clostridia_o;Oscillospirales_f;Ruminococcaceae;_ | 0.06 ± 0.08 | 0.21 ± 0.60 |
| d;Bacteria_p;Firmicutes_c;Clostridia_o;Clostridia_UCG_014_f;Clostridia_UCG_014_g;Clostridia_UCG_014 | 1.41 ± 2.16 | 1.62 ± 2.87 |
| d;Bacteria_p;Proteobacteria_c;Gammaproteobacteria_o;Enterobacterales_f;Enterobacteriaceae_g;Escherichia_Shigella | 1.17 ± 2.41 | 1.23 ± 2.21 |
| d;Bacteria_p;Firmicutes_c;Clostridia_o;Oscillospirales_f;Ruminococcaceae_g;Incertae_Sedis | 0.29 ± 0.41 | 0.53 ± 0.69 |
| d;Bacteria_p;Firmicutes_c;Bacilli_o;Lactobacillales_f;Lactobacillaceae_g;Lactobacillus | 0.47 ± 1.06 | 0.31 ± 1.04 |
| d;Bacteria_p;Bacteroidota_c;Bacteroidia_o;Bacteroidales_f;Prevotellaceae_g;Prevotella | 14.04 ± 20.11 | 8.11 ± 10.26 |
| d;Bacteria_p;Actinobacteriota_c;Coriobacteriia_o;Coriobacteriales_f;uncultured_g;uncultured | 0.00 ± 0.00 | 0.00 ± 0.01 |
| d;Bacteria_p;Firmicutes_c;Clostridia_o;Christensenellales_f;Christensenellaceae_g;Christensenellaceae_R_7_group | 0.85 ± 1.33 | 1.67 ± 3.14 |
| d;Bacteria_p;Actinobacteriota_c;Coriobacteriia_o;Coriobacteriales_f;Eggerthellaceae_g;uncultured | 0.00 ± 0.01 | 0.03 ± 0.08 |
| d;Bacteria_p;Bacteroidota_c;Bacteroidia_o;Bacteroidales_f;Marinifilaceae_g;Butyricimonas | 0.11 ± 0.28 | 0.10 ± 0.18 |
| d;Bacteria_p;Firmicutes_c;Clostridia_o;Oscillospirales_f;Oscillospiraceae_g;Oscillibacter | 0.23 ± 0.46 | 0.44 ± 0.73 |
| d;Bacteria_p;Firmicutes_c;Clostridia_o;Lachnospirales_f;Lachnospiraceae_g;uncultured | 0.08 ± 0.19 | 0.14 ± 0.34 |
| d;Bacteria_p;Firmicutes_c;Clostridia_o;Oscillospirales_f;Ruminococcaceae_g;Faecalibacterium | 6.82 ± 3.96 | 5.01 ± 4.79 |
| d;Bacteria_p;Firmicutes_c;Clostridia_o;Lachnospirales_f;Lachnospiraceae_g;Marvinbryantia | 0.00 ± 0.00 | 0.01 ± 0.01 |
| d;Bacteria_p;Firmicutes_c;Clostridia_o;Lachnospirales_f;Lachnospiraceae_g;Coprococcus | 0.76 ± 1.80 | 1.14 ± 1.99 |
| d;Bacteria_p;Firmicutes_c;Negativicutes_o;Veillonellales_Selenomonadales_f;Veillonellaceae_g;Megasphaera | 0.35 ± 1.15 | 0.43 ± 1.09 |
| d;Bacteria_p;Actinobacteriota_c;Coriobacteriia_o;Coriobacteriales_f;Eggerthellaceae_g;Senegalimassilia | 0.03 ± 0.13 | 0.02 ± 0.04 |
| d;Bacteria_p;Firmicutes_c;Clostridia_o;Oscillospirales_f;Ruminococcaceae_g;uncultured | 0.31 ± 0.96 | 0.38 ± 0.69 |
| d;Bacteria_p;Patescibacteria_c;Saccharimonadia_o;Saccharimonadales_f;Saccharimonadaceae;_ | 0.00 ± 0.00 | 0.00 ± 0.01 |
| d;Bacteria_p;Firmicutes_c;Negativicutes_o;Acidaminococcales_f;Acidaminococcaceae_g;Phascolarctobacterium | 1.64 ± 2.31 | 1.61 ± 1.43 |
| d;Bacteria_p;Firmicutes_c;Clostridia_o;Oscillospirales_f;Ruminococcaceae_g;Ruminococcus | 0.74 ± 0.76 | 1.02 ± 1.19 |
| d;Bacteria_p;Firmicutes_c;Clostridia_o;Oscillospirales_f;_Eubacterium;coprostanoligenes_group_g;_Eubacterium;coprostanoligenes_group | 1.08 ± 1.42 | 1.66 ± 2.98 |
| d;Bacteria_p;Actinobacteriota_c;Actinobacteria_o;Bifidobacteriales_f;Bifidobacteriaceae_g;Bifidobacterium | 2.75 ± 3.70 | 3.32 ± 4.05 |
| d;Bacteria_p;Firmicutes_c;Bacilli_o;Erysipelotrichales_f;Erysipelotrichaceae_g;Erysipelotrichaceae_UCG_006 | 0.00 ± 0.00 | 0.00 ± 0.00 |
| d;Bacteria_p;Firmicutes_c;Clostridia_o;Lachnospirales_f;Lachnospiraceae_g;Dorea | 0.30 ± 0.33 | 0.38 ± 0.39 |
| d;Bacteria_p;Proteobacteria_c;Gammaproteobacteria_o;Burkholderiales;_ | 0.01 ± 0.04 | 0.00 ± 0.01 |
| d;Bacteria_p;Actinobacteriota_c;Coriobacteriia_o;Coriobacteriales_f;Atopobiaceae_g;Olsenella | 0.00 ± 0.00 | 0.04 ± 0.19 |
| d;Bacteria_p;Firmicutes_c;Clostridia_o;Lachnospirales_f;Lachnospiraceae_g;Eubacterium;ventriosum_group | 0.29 ± 0.42 | 0.26 ± 0.32 |
| d;Bacteria_p;Firmicutes_c;Clostridia_o;Oscillospirales_f;Oscillospiraceae;_ | 0.15 ± 0.28 | 0.15 ± 0.14 |
| d;Bacteria_p;Firmicutes_c;Clostridia_o;Oscillospirales_f;Oscillospiraceae_g;UCG_003 | 0.12 ± 0.17 | 0.15 ± 0.15 |
| d;Bacteria_p;Firmicutes_c;Clostridia_o;Oscillospirales_f;_Clostridium;methylpentosum_group_g;_Clostridium;methylpentosum_group | 0.01 ± 0.03 | 0.01 ± 0.01 |
| d;Bacteria_p;Firmicutes_c;Bacilli_o;Erysipelotrichales_f;Erysipelatoclostridiaceae_g;Erysipelotrichaceae_UCG_003 | 0.41 ± 0.75 | 0.28 ± 0.39 |
| d;Bacteria_p;Bacteroidota_c;Bacteroidia_o;Bacteroidales_f;Tannerellaceae_g;Parabacteroides | 2.10 ± 1.94 | 2.05 ± 1.40 |
| d;Bacteria_p;Firmicutes_c;Clostridia_o;Monoglobales_f;Monoglobaceae_g;Monoglobus | 0.25 ± 0.24 | 0.50 ± 0.69 |
| d;Bacteria;_;_;_;_ | 0.01 ± 0.02 | 0.01 ± 0.02 |
| d;Bacteria_p;Bacteroidota_c;Bacteroidia;_;__ | 0.00 ± 0.00 | 0.00 ± 0.00 |
| d;Bacteria_p;Firmicutes_c;Negativicutes_o;Veillonellales_Selenomonadales_f;Veillonellaceae_g;Dialister | 1.68 ± 4.26 | 2.18 ± 4.18 |
| d;Bacteria_p;Firmicutes_c;Clostridia_o;Clostridiales_f;Clostridiaceae_g;Clostridium_sensu_stricto_1 | 0.06 ± 0.17 | 0.11 ± 0.26 |
| d;Bacteria_p;Actinobacteriota_c;Coriobacteriia_o;Coriobacteriales_f;Eggerthellaceae_g;Adlercreutzia | 0.00 ± 0.01 | 0.00 ± 0.01 |
| d;Bacteria_p;Firmicutes_c;Clostridia_o;Oscillospirales_f;Oscillospiraceae_g;Colidextribacter | 0.08 ± 0.26 | 0.22 ± 0.50 |
| d;Bacteria_p;Actinobacteriota_c;Coriobacteriia_o;Coriobacteriales_f;Coriobacteriaceae_g;Collinsella | 0.57 ± 1.43 | 0.47 ± 0.90 |
| d;Bacteria_p;Verrucomicrobiota_c;Lentisphaeria_o;Victivallales_f;Victivallaceae_g;Victivallis | 0.00 ± 0.00 | 0.00 ± 0.00 |
| d;Bacteria_p;Firmicutes_c;Clostridia_o;Peptostreptococcales_Tissierellales_f;Anaerovoracaceae_g;Family_XIII_AD3011_group | 0.03 ± 0.06 | 0.04 ± 0.04 |
| d;Bacteria_p;Firmicutes_c;Clostridia_o;Oscillospirales_f;Ruminococcaceae_g;DTU089 | 0.00 ± 0.01 | 0.03 ± 0.05 |
| d;Bacteria_p;Bacteroidota_c;Bacteroidia_o;Bacteroidales;_; | 0.39 ± 0.68 | 0.92 ± 1.89 |
| d;Bacteria_p;Verrucomicrobiota_c;Verrucomicrobiae_o;Verrucomicrobiales_f;Akkermansiaceae_g;Akkermansia | 0.00 ± 0.01 | 0.10 ± 0.33 |
| d;Bacteria_p;Bacteroidota_c;Bacteroidia_o;Bacteroidales_f;Prevotellaceae_g;Alloprevotella | 0.40 ± 0.88 | 0.61 ± 1.32 |
| d;Bacteria_p;Firmicutes_c;Bacilli_o;Lactobacillales_f;Streptococcaceae_g;Streptococcus | 1.12 ± 2.64 | 0.83 ± 2.12 |
| d;Bacteria_p;Firmicutes_c;Clostridia_o;Oscillospirales;_; | 0.01 ± 0.03 | 0.03 ± 0.06 |
| d;Bacteria_p;Firmicutes_c;Clostridia_o;Lachnospirales_f;Lachnospiraceae_g;Lachnoclostridium | 1.16 ± 1.91 | 0.92 ± 0.74 |
| d;Bacteria_p;Firmicutes_c;Negativicutes_o;Veillonellales_Selenomonadales_f;Veillonellaceae_g;Negativicoccus | 0.00 ± 0.00 | 0.00 ± 0.00 |
| d;Bacteria_p;Bacteroidota_c;Bacteroidia_o;Bacteroidales_f;Tannerellaceae;_ | 0.03 ± 0.07 | 0.04 ± 0.06 |
| d;Bacteria_p;Firmicutes_c;Clostridia_o;Lachnospirales_f;Lachnospiraceae_g;Anaerostipes | 0.36 ± 0.61 | 0.16 ± 0.21 |
| d;Bacteria_p;Firmicutes_c;Negativicutes_o;Acidaminococcales_f;Acidaminococcaceae_g;Succiniclasticum | 0.00 ± 0.00 | 0.00 ± 0.00 |
| d;Bacteria_p;Firmicutes_c;Clostridia_o;Oscillospirales_f;Ruminococcaceae_g;Paludicola | 0.00 ± 0.01 | 0.02 ± 0.03 |
| d;Bacteria_p;Fusobacteriota_c;Fusobacteriia_o;Fusobacteriales_f;Leptotrichiaceae_g;Leptotrichia | 0.01 ± 0.01 | 0.00 ± 0.00 |
| d;Bacteria_p;Firmicutes_c;Clostridia_o;Oscillospirales_f;Butyricicoccaceae_g;Butyricicoccus | 0.25 ± 0.25 | 0.30 ± 0.24 |
| d;Bacteria_p;Firmicutes_c;Clostridia_o;Lachnospirales_f;Lachnospiraceae_g;Catenibacillus | 0.00 ± 0.00 | 0.00 ± 0.01 |
| d;Bacteria_p;Fusobacteriota_c;Fusobacteriia_o;Fusobacteriales_f;Leptotrichiaceae_g;Sneathia | 0.00 ± 0.00 | 0.00 ± 0.00 |
| d;Bacteria_p;Firmicutes_c;Negativicutes_o;Veillonellales_Selenomonadales_f;Selenomonadaceae;_ | 0.00 ± 0.00 | 0.00 ± 0.00 |
| d;Bacteria_p;Actinobacteriota_c;Coriobacteriia_o;Coriobacteriales_f;Eggerthellaceae;_ | 0.00 ± 0.00 | 0.00 ± 0.00 |
| d;Bacteria_p;Firmicutes_c;Negativicutes_o;Veillonellales_Selenomonadales_f;Veillonellaceae_g;Allisonella | 0.03 ± 0.09 | 0.05 ± 0.10 |
| d;Bacteria_p;Firmicutes_c;Clostridia_o;Oscillospirales_f;Oscillospiraceae_g;Flavonifractor | 0.23 ± 0.40 | 0.26 ± 0.43 |
| d;Bacteria_p;Firmicutes_c;Clostridia_o;Lachnospirales_f;Lachnospiraceae_g;Lachnospiraceae_UCG_004 | 0.02 ± 0.02 | 0.02 ± 0.03 |
| d;Bacteria_p;Bacteroidota_c;Bacteroidia_o;Bacteroidales_f;Muribaculaceae_g;Muribaculaceae | 0.11 ± 0.54 | 0.23 ± 1.12 |
| d;Bacteria_p;Firmicutes_c;Clostridia_o;Lachnospirales_f;Lachnospiraceae_g;Agathobacter | 2.48 ± 4.40 | 2.89 ± 4.86 |
| d;Bacteria_p;Firmicutes_c;Clostridia_o;Lachnospirales_f;Lachnospiraceae_g;_Eubacterium;eligens_group | 1.28 ± 2.41 | 0.56 ± 0.67 |
| d;Bacteria_p;Proteobacteria_c;Gammaproteobacteria_o;Burkholderiales_f;Comamonadaceae_g;Comamonas | 0.00 ± 0.00 | 0.00 ± 0.00 |
| d;Bacteria_p;Firmicutes_c;Clostridia_o;Clostridia_vadinBB60_group_f;Clostridia_vadinBB60_group_g;Clostridia_vadinBB60_group | 0.01 ± 0.03 | 0.02 ± 0.04 |
| d;Bacteria_p;Firmicutes_c;Clostridia_o;Oscillospirales_f;Oscillospiraceae_g;UCG_005 | 0.32 ± 0.56 | 0.79 ± 1.89 |
| d;Bacteria_p;Verrucomicrobiota_c;Lentisphaeria_o;Victivallales_f;vadinBE97_g;vadinBE97 | 0.00 ± 0.01 | 0.00 ± 0.00 |
| d;Bacteria_p;Firmicutes_c;Bacilli_o;Lactobacillales_f;Enterococcaceae_g;Enterococcus | 0.02 ± 0.07 | 0.01 ± 0.03 |
| d;Bacteria_p;Firmicutes_c;Clostridia_o;Oscillospirales_f;Oscillospiraceae_g;UCG_002 | 1.23 ± 2.00 | 1.75 ± 1.63 |
| d;Bacteria_p;Proteobacteria_c;Gammaproteobacteria_o;Burkholderiales_f;Oxalobacteraceae_g;Oxalobacter | 0.00 ± 0.01 | 0.02 ± 0.05 |
| d;Bacteria_p;Proteobacteria_c;Gammaproteobacteria_o;Enterobacterales_f;Enterobacteriaceae_g;Klebsiella | 0.08 ± 0.17 | 0.09 ± 0.29 |
| d;Bacteria_p;Verrucomicrobiota_c;Lentisphaeria_o;Victivallales_f;Victivallaceae_g;Victivallaceae | 0.00 ± 0.00 | 0.00 ± 0.00 |
| d;Bacteria_p;Desulfobacterota_c;Desulfovibrionia_o;Desulfovibrionales_f;Desulfovibrionaceae_g;Desulfovibrio | 0.02 ± 0.06 | 0.11 ± 0.26 |
| d;Bacteria_p;Firmicutes_c;Clostridia_o;Oscillospirales_f;Ruminococcaceae_g;Negativibacillus | 0.02 ± 0.08 | 0.23 ± 0.74 |
| d;Bacteria_p;Firmicutes_c;Clostridia_o;Oscillospirales_f;UCG_010_g;UCG_010 | 0.09 ± 0.22 | 0.18 ± 0.38 |
| d;Bacteria_p;Fusobacteriota_c;Fusobacteriia_o;Fusobacteriales_f;Fusobacteriaceae_g;Fusobacterium | 0.04 ± 0.10 | 0.23 ± 0.95 |
| d;Bacteria_p;Bacteroidota_c;Bacteroidia_o;Bacteroidales_f;Marinifilaceae_g;Odoribacter | 0.09 ± 0.11 | 0.13 ± 0.15 |
| d;Bacteria_p;Firmicutes_c;Clostridia_o;Lachnospirales_f;Lachnospiraceae_g;Anaerosporobacter | 0.00 ± 0.01 | 0.01 ± 0.03 |
| d;Bacteria_p;Firmicutes_c;Negativicutes_o;Veillonellales_Selenomonadales_f;Veillonellaceae_g;Veillonella | 1.67 ± 4.07 | 0.71 ± 2.39 |
| d;Bacteria_p;Firmicutes_c;Clostridia_o;Lachnospirales_f;Lachnospiraceae_g;Moryella | 0.02 ± 0.06 | 0.07 ± 0.13 |
| d;Bacteria_p;Firmicutes_c;Bacilli_o;Erysipelotrichales_f;Erysipelotrichaceae_g;Dielma | 0.00 ± 0.00 | 0.00 ± 0.00 |
| d;Bacteria_p;Patescibacteria_c;Saccharimonadia_o;Saccharimonadales_f;Saccharimonadaceae_g;Candidatus_Saccharimonas | 0.00 ± 0.00 | 0.00 ± 0.00 |
| d;Bacteria_p;Bacteroidota_c;Bacteroidia_o;Bacteroidales_f;Porphyromonadaceae_g;Porphyromonas | 0.00 ± 0.01 | 0.00 ± 0.02 |
| d;Bacteria_p;Firmicutes_c;Bacilli_o;RF39_f;RF39_g;RF39 | 0.02 ± 0.06 | 0.16 ± 0.49 |
| d;Bacteria_p;Firmicutes_c;Clostridia_o;Oscillospirales_f;Oscillospiraceae_g;Oscillospira | 0.00 ± 0.01 | 0.01 ± 0.03 |
| d;Bacteria_p;Firmicutes_c;Clostridia_o;Lachnospirales_f;Lachnospiraceae_g;Lachnospiraceae_UCG_001 | 0.04 ± 0.12 | 0.02 ± 0.08 |
| d;Bacteria_p;Actinobacteriota_c;Coriobacteriia_o;Coriobacteriales_f;Eggerthellaceae_g;Slackia | 0.01 ± 0.02 | 0.01 ± 0.03 |
| d;Bacteria_p;Firmicutes_c;Clostridia_o;Lachnospirales_f;Lachnospiraceae_g;Lachnospiraceae_FCS020_group | 0.02 ± 0.08 | 0.01 ± 0.02 |
| d;Bacteria_p;Actinobacteriota;_;_;_ | 0.00 ± 0.00 | 0.00 ± 0.00 |
| d;Bacteria_p;Firmicutes_c;Bacilli_o;Erysipelotrichales_f;Erysipelatoclostridiaceae_g;Catenibacterium | 0.10 ± 0.28 | 0.41 ± 0.96 |
| d;Bacteria_p;Firmicutes_c;Clostridia_o;Peptostreptococcales_Tissierellales_f;Peptostreptococcaceae_g;Paraclostridium | 0.00 ± 0.00 | 0.00 ± 0.00 |
| d;Bacteria_p;Firmicutes_c;Clostridia_o;Lachnospirales_f;Lachnospiraceae_g;Sellimonas | 0.01 ± 0.05 | 0.02 ± 0.08 |
| d;Bacteria_p;Firmicutes_c;Clostridia_o;Oscillospirales_f;Oscillospiraceae_g;NK4A214_group | 0.19 ± 0.37 | 0.39 ± 0.46 |
| d;Bacteria_p;Firmicutes_c;Negativicutes_o;Veillonellales_Selenomonadales_f;Selenomonadaceae_g;Mitsuokella | 0.28 ± 0.86 | 0.34 ± 0.85 |
| d;Bacteria_p;Firmicutes_c;Bacilli_o;Erysipelotrichales_f;Erysipelotrichaceae_g;Holdemanella | 1.30 ± 2.77 | 1.04 ± 2.60 |
| d;Bacteria_p;Firmicutes_c;Bacilli_o;Lactobacillales_f;Carnobacteriaceae_g;Granulicatella | 0.03 ± 0.03 | 0.01 ± 0.02 |
| d;Bacteria_p;Proteobacteria_c;Gammaproteobacteria_o;Pasteurellales_f;Pasteurellaceae_g;Aggregatibacter | 0.01 ± 0.04 | 0.00 ± 0.00 |
| d;Bacteria_p;Firmicutes_c;Bacilli_o;Lactobacillales_f;Streptococcaceae_g;Lactococcus | 0.00 ± 0.01 | 0.00 ± 0.00 |
| d;Bacteria_p;Firmicutes_c;Clostridia_o;Peptostreptococcales_Tissierellales_f;Peptostreptococcales_Tissierellales_g;Anaerococcus | 0.00 ± 0.01 | 0.00 ± 0.00 |
| d;Bacteria_p;Firmicutes_c;Clostridia_o;Oscillospirales_f;Oscillospiraceae_g;uncultured | 0.11 ± 0.13 | 0.23 ± 0.26 |
| d;Bacteria_p;Proteobacteria_c;Gammaproteobacteria_o;Aeromonadales_f;Succinivibrionaceae_g;Succinivibrio | 0.00 ± 0.00 | 0.00 ± 0.01 |
| d;Bacteria_p;Bacteroidota_c;Bacteroidia_o;Bacteroidales_f;Rikenellaceae_g;Rikenellaceae_RC9_gut_group | 0.03 ± 0.10 | 0.05 ± 0.21 |
| d;Bacteria_p;Bacteroidota_c;Bacteroidia_o;Bacteroidales_f;Tannerellaceae_g;Tannerellaceae | 0.00 ± 0.00 | 0.00 ± 0.01 |
| d;Bacteria_p;Firmicutes_c;Clostridia_o;Oscillospirales_f;Butyricicoccaceae_g;UCG_009 | 0.01 ± 0.03 | 0.03 ± 0.05 |
| d;Bacteria_p;Firmicutes_c;Clostridia_o;Peptostreptococcales_Tissierellales_f;Peptostreptococcaceae;_ | 0.02 ± 0.08 | 0.01 ± 0.01 |
| d;Bacteria_p;Bacteroidota_c;Bacteroidia_o;Bacteroidales_f;Dysgonomonadaceae_g;Dysgonomonas | 0.00 ± 0.02 | 0.05 ± 0.24 |
| d;Bacteria_p;Firmicutes_c;Clostridia_o;Lachnospirales_f;Lachnospiraceae_g;Roseburia | 0.68 ± 0.91 | 1.19 ± 1.15 |
| d;Bacteria_p;Firmicutes_c;Clostridia_o;Lachnospirales_f;Lachnospiraceae_g;_Eubacterium;hallii_group | 0.05 ± 0.08 | 0.05 ± 0.07 |
| d;Bacteria_p;Firmicutes_c;Clostridia_o;Lachnospirales_f;Lachnospiraceae_g;Eisenbergiella | 0.00 ± 0.00 | 0.04 ± 0.14 |
| d;Bacteria_p;Proteobacteria_c;Gammaproteobacteria_o;Pseudomonadales_f;Moraxellaceae_g;Acinetobacter | 0.00 ± 0.00 | 0.00 ± 0.00 |
| d;Bacteria_p;Firmicutes;_;_;_ | 0.00 ± 0.01 | 0.01 ± 0.01 |
| d;Bacteria_p;Bacteroidota_c;Bacteroidia_o;Bacteroidales_f;Prevotellaceae;_ | 0.15 ± 0.54 | 0.35 ± 1.29 |
| d;Bacteria_p;Firmicutes_c;Bacilli_o;Erysipelotrichales_f;Erysipelotrichaceae_g;_Clostridium;innocuum_group | 0.02 ± 0.06 | 0.01 ± 0.01 |
| d;Bacteria_p;Firmicutes_c;Clostridia_o;Lachnospirales_f;Lachnospiraceae_g;Butyrivibrio | 0.00 ± 0.01 | 0.01 ± 0.04 |
| d;Bacteria_p;Actinobacteriota_c;Actinobacteria_o;Bifidobacteriales_f;Bifidobacteriaceae_g;Gardnerella | 0.00 ± 0.00 | 0.00 ± 0.01 |
| d;Bacteria_p;Firmicutes_c;Clostridia_o;Oscillospirales_f;Ruminococcaceae_g;Anaerotruncus | 0.00 ± 0.01 | 0.02 ± 0.03 |
| d;Bacteria_p;Firmicutes_c;Clostridia_o;Lachnospirales_f;Lachnospiraceae_g;Lachnospiraceae_NK4A136_group | 0.77 ± 1.48 | 1.40 ± 2.65 |
| d;Bacteria_p;Firmicutes_c;Clostridia_o;Lachnospirales_f;Lachnospiraceae_g;_Ruminococcus;torques_group | 0.25 ± 0.33 | 0.48 ± 1.51 |
| d;Bacteria_p;Proteobacteria_c;Alphaproteobacteria_o;Rhodospirillales_f;uncultured_g;uncultured | 0.00 ± 0.00 | 0.00 ± 0.00 |
| d;Bacteria_p;Firmicutes_c;Clostridia_o;Oscillospirales_f;Ruminococcaceae_g;Anaerofilum | 0.00 ± 0.00 | 0.00 ± 0.01 |
| d;Bacteria_p;Desulfobacterota_c;Desulfovibrionia_o;Desulfovibrionales_f;Desulfovibrionaceae_g;Bilophila | 0.17 ± 0.26 | 0.24 ± 0.30 |
| d;Bacteria_p;Firmicutes_c;Clostridia_o;Oscillospirales_f;Ruminococcaceae_g;_Eubacterium;siraeum_group | 0.18 ± 0.39 | 0.13 ± 0.24 |
| d;Bacteria_p;Firmicutes_c;Clostridia_o;Oscillospirales_f;Ruminococcaceae_g;Phocea | 0.00 ± 0.00 | 0.01 ± 0.01 |
| d;Bacteria_p;Firmicutes_c;Clostridia_o;Peptostreptococcales_Tissierellales_f;Peptostreptococcales_Tissierellales_g;Peptoniphilus | 0.00 ± 0.01 | 0.00 ± 0.01 |
| d;Bacteria_p;Firmicutes_c;Clostridia_o;Lachnospirales_f;Lachnospiraceae_g;Frisingicoccus | 0.00 ± 0.00 | 0.01 ± 0.03 |
| d;Bacteria_p;Cyanobacteria_c;Vampirivibrionia_o;Gastranaerophilales_f;Gastranaerophilales_g;Gastranaerophilales | 0.00 ± 0.02 | 0.00 ± 0.00 |
| d;Bacteria_p;Firmicutes_c;Clostridia_o;Oscillospirales_f;Oscillospiraceae_g;Intestinimonas | 0.00 ± 0.01 | 0.00 ± 0.01 |
| d;Bacteria_p;Campilobacterota_c;Campylobacteria_o;Campylobacterales_f;Campylobacteraceae_g;Campylobacter | 0.01 ± 0.02 | 0.00 ± 0.01 |
| d;Bacteria_p;Firmicutes_c;Clostridia_o;Lachnospirales_f;Lachnospiraceae_g;Lachnospiraceae | 0.02 ± 0.06 | 0.02 ± 0.05 |
| d;Bacteria_p;Firmicutes_c;Clostridia_o;Lachnospirales_f;Defluviitaleaceae_g;Defluviitaleaceae_UCG_011 | 0.01 ± 0.02 | 0.02 ± 0.03 |
| d;Bacteria_p;Actinobacteriota_c;Coriobacteriia_o;Coriobacteriales_f;Coriobacteriales_Incertae_Sedis_g;uncultured | 0.00 ± 0.01 | 0.01 ± 0.02 |
| d;Bacteria_p;Proteobacteria_c;Gammaproteobacteria_o;Burkholderiales_f;Neisseriaceae_g;Neisseria | 0.01 ± 0.02 | 0.01 ± 0.03 |
| d;Bacteria_p;Firmicutes_c;Clostridia_o;Lachnospirales_f;Lachnospiraceae_g;_Eubacterium;ruminantium_group | 0.92 ± 3.85 | 0.89 ± 2.30 |
| d;Bacteria_p;Firmicutes_c;Bacilli_o;Erysipelotrichales_f;Erysipelotrichaceae_g;Holdemania | 0.01 ± 0.03 | 0.06 ± 0.12 |
| d;Bacteria_p;Firmicutes_c;Bacilli_o;Erysipelotrichales_f;Erysipelotrichaceae_g;Turicibacter | 0.00 ± 0.01 | 0.01 ± 0.02 |
| d;Bacteria_p;Bacteroidota_c;Bacteroidia_o;Flavobacteriales_f;Flavobacteriaceae_g;uncultured | 0.00 ± 0.01 | 0.00 ± 0.01 |
| d;Bacteria_p;Firmicutes_c;Clostridia_o;Peptostreptococcales_Tissierellales_f;Peptostreptococcaceae_g;Romboutsia | 0.34 ± 1.11 | 0.62 ± 2.68 |
| d;Bacteria_p;Firmicutes_c;Clostridia_o;Oscillospirales_f;Ruminococcaceae_g;Fournierella | 0.01 ± 0.02 | 0.02 ± 0.08 |
| d;Bacteria_p;Firmicutes_c;Clostridia_o;Christensenellales_f;Christensenellaceae_g;uncultured | 0.01 ± 0.01 | 0.02 ± 0.05 |
| d;Bacteria_p;Firmicutes_c;Clostridia_o;Lachnospirales_f;Lachnospiraceae_g;_Eubacterium;oxidoreducens_group | 0.00 ± 0.01 | 0.01 ± 0.02 |
| d;Bacteria_p;Synergistota_c;Synergistia_o;Synergistales_f;Synergistaceae_g;Cloacibacillus | 0.00 ± 0.00 | 0.00 ± 0.00 |
| d;Bacteria_p;Firmicutes_c;Clostridia_o;Christensenellales_f;Christensenellaceae_g;Christensenella | 0.00 ± 0.00 | 0.01 ± 0.02 |
| d;Bacteria_p;Firmicutes_c;Clostridia_o;Peptostreptococcales_Tissierellales_f;Peptostreptococcales_Tissierellales_g;Fenollaria | 0.00 ± 0.00 | 0.00 ± 0.01 |
| d;Bacteria_p;Firmicutes_c;Clostridia_o;Christensenellales_f;Christensenellaceae_g;Catabacter | 0.00 ± 0.01 | 0.00 ± 0.01 |
| d;Bacteria_p;Firmicutes_c;Clostridia_o;Peptostreptococcales_Tissierellales_f;Peptostreptococcaceae_g;uncultured | 0.00 ± 0.00 | 0.00 ± 0.00 |
| d;Bacteria_p;Firmicutes_c;Bacilli_o;Erysipelotrichales_f;Erysipelatoclostridiaceae_g;Coprobacillus | 0.00 ± 0.02 | 0.00 ± 0.01 |
| d;Bacteria_p;Firmicutes_c;Bacilli_o;Lactobacillales_f;Leuconostocaceae_g;Weissella | 0.01 ± 0.02 | 0.02 ± 0.07 |
| d;Bacteria_p;Proteobacteria_c;Gammaproteobacteria_o;Pasteurellales_f;Pasteurellaceae;_ | 0.00 ± 0.00 | 0.00 ± 0.00 |
| d;Bacteria_p;Firmicutes_c;Clostridia_o;Lachnospirales;_; | 0.00 ± 0.00 | 0.00 ± 0.00 |
| d;Bacteria_p;Firmicutes_c;Clostridia_o;Oscillospirales_f;Ruminococcaceae_g;Angelakisella | 0.00 ± 0.00 | 0.00 ± 0.00 |
| d;Bacteria_p;Firmicutes_c;uncultured_o;uncultured_f;uncultured_g;uncultured | 0.00 ± 0.00 | 0.00 ± 0.00 |
| d;Bacteria_p;Firmicutes_c;Negativicutes_o;Acidaminococcales_f;Acidaminococcaceae_g;Acidaminococcus | 0.26 ± 1.23 | 0.61 ± 2.43 |
| d;Bacteria_p;Firmicutes_c;Clostridia_o;Lachnospirales_f;Lachnospiraceae_g;Hungatella | 0.07 ± 0.23 | 0.06 ± 0.15 |
| d;Bacteria_p;Firmicutes_c;Negativicutes_o;Veillonellales_Selenomonadales_f;Selenomonadaceae_g;Selenomonas | 0.00 ± 0.00 | 0.00 ± 0.00 |
| d;Bacteria_p;Firmicutes_c;Clostridia_o;Oscillospirales_f;uncultured_g;uncultured | 0.00 ± 0.00 | 0.00 ± 0.00 |
| d;Bacteria_p;Actinobacteriota_c;Coriobacteriia_o;Coriobacteriales_f;Atopobiaceae_g;Libanicoccus | 0.00 ± 0.00 | 0.00 ± 0.01 |
| d;Bacteria_p;Firmicutes_c;Clostridia_o;Lachnospirales_f;Lachnospiraceae_g;Tuzzerella | 0.00 ± 0.00 | 0.00 ± 0.00 |
| d;Bacteria_p;Actinobacteriota_c;Actinobacteria_o;Actinomycetales_f;Actinomycetaceae_g;Actinomyces | 0.02 ± 0.04 | 0.01 ± 0.01 |
| d;Bacteria_p;Firmicutes_c;Bacilli_o;Bacillales_f;Bacillaceae_g;Bacillus | 0.00 ± 0.00 | 0.00 ± 0.00 |
| d;Bacteria_p;Firmicutes_c;Clostridia_o;Eubacteriales_f;Anaerofustaceae_g;Anaerofustis | 0.00 ± 0.00 | 0.00 ± 0.00 |
| d;Bacteria_p;Firmicutes_c;Clostridia_o;Lachnospirales_f;Lachnospiraceae_g;Tyzzerella | 0.10 ± 0.27 | 0.17 ± 0.56 |
| d;Bacteria_p;Proteobacteria_c;Gammaproteobacteria_o;Cardiobacteriales_f;Cardiobacteriaceae_g;Cardiobacterium | 0.00 ± 0.00 | 0.00 ± 0.00 |
| d;Bacteria_p;Proteobacteria_c;Gammaproteobacteria_o;Enterobacterales_f;Morganellaceae_g;Morganella | 0.00 ± 0.01 | 0.00 ± 0.00 |
| d;Bacteria_p;Verrucomicrobiota_c;Verrucomicrobiae_o;Opitutales_f;Puniceicoccaceae_g;uncultured | 0.00 ± 0.02 | 0.00 ± 0.01 |
| d;Bacteria_p;Firmicutes_c;Clostridia_o;Oscillospirales_f;Ruminococcaceae_g;CAG_352 | 0.01 ± 0.03 | 0.55 ± 1.90 |
| d;Bacteria_p;Bacteroidota_c;Bacteroidia_o;Bacteroidales_f;uncultured_g;uncultured | 0.00 ± 0.00 | 0.00 ± 0.00 |
| d;Bacteria_p;Firmicutes_c;Clostridia_o;Peptostreptococcales_Tissierellales_f;Anaerovoracaceae_g;Family_XIII_UCG_001 | 0.02 ± 0.03 | 0.04 ± 0.05 |
| d;Bacteria_p;Proteobacteria_c;Gammaproteobacteria;_;__ | 0.00 ± 0.01 | 0.00 ± 0.00 |
| d;Bacteria_p;Firmicutes_c;Clostridia_o;Lachnospirales_f;Lachnospiraceae_g;Fusicatenibacter | 0.17 ± 0.18 | 0.21 ± 0.25 |
| d;Bacteria_p;Firmicutes_c;Clostridia_o;Peptostreptococcales_Tissierellales_f;Peptostreptococcales_Tissierellales_g;Finegoldia | 0.00 ± 0.01 | 0.00 ± 0.00 |
| d;Bacteria_p;Patescibacteria_c;Saccharimonadia_o;Saccharimonadales_f;Saccharimonadales_g;Saccharimonadales | 0.00 ± 0.00 | 0.00 ± 0.00 |
| d;Bacteria_p;Firmicutes_c;Clostridia_o;Oscillospirales_f;Ruminococcaceae_g;Harryflintia | 0.00 ± 0.01 | 0.00 ± 0.01 |
| d;Bacteria_p;Firmicutes_c;Clostridia_o;Christensenellales_f;Christensenellaceae_g;Christensenellaceae | 0.00 ± 0.00 | 0.00 ± 0.00 |
| d;Bacteria_p;Bacteroidota_c;Bacteroidia_o;Bacteroidales_f;Prevotellaceae_g;uncultured | 0.00 ± 0.01 | 0.00 ± 0.00 |
| d;Bacteria_p;Firmicutes_c;Clostridia_o;Clostridia_f;Hungateiclostridiaceae_g;Fastidiosipila | 0.00 ± 0.00 | 0.00 ± 0.00 |
| d;Bacteria_p;Proteobacteria_c;Gammaproteobacteria_o;Enterobacterales_f;Enterobacteriaceae_g;Enterobacter | 0.00 ± 0.02 | 0.00 ± 0.00 |
| d;Bacteria_p;Firmicutes_c;Clostridia_o;Oscillospirales_f;Butyricicoccaceae;_ | 0.00 ± 0.00 | 0.00 ± 0.01 |
| d;Bacteria_p;Bacteroidota_c;Bacteroidia_o;Bacteroidales_f;Prevotellaceae_g;Prevotellaceae_NK3B31_group | 0.01 ± 0.05 | 0.00 ± 0.01 |
| d;Bacteria_p;Firmicutes_c;Clostridia_o;Peptococcales_f;Peptococcaceae_g;Peptococcus | 0.00 ± 0.00 | 0.00 ± 0.01 |
| d;Bacteria_p;Firmicutes_c;Bacilli_o;Erysipelotrichales_f;Erysipelatoclostridiaceae_g;Candidatus_Stoquefichus | 0.00 ± 0.00 | 0.00 ± 0.00 |
| d;Bacteria_p;Firmicutes_c;Bacilli_o;Erysipelotrichales_f;Erysipelatoclostridiaceae_g;Asteroleplasma | 0.00 ± 0.00 | 0.00 ± 0.00 |
| d;Bacteria_p;Synergistota_c;Synergistia_o;Synergistales_f;Synergistaceae;_ | 0.00 ± 0.00 | 0.00 ± 0.00 |
| d;Bacteria_p;Firmicutes_c;Clostridia_o;Lachnospirales_f;Lachnospiraceae_g;Oribacterium | 0.00 ± 0.00 | 0.00 ± 0.01 |
| d;Bacteria_p;Desulfobacterota_c;Desulfovibrionia_o;Desulfovibrionales_f;Desulfovibrionaceae;_ | 0.00 ± 0.00 | 0.00 ± 0.01 |
| d;Bacteria_p;Proteobacteria_c;Gammaproteobacteria_o;Pseudomonadales_f;Moraxellaceae_g;Enhydrobacter | 0.00 ± 0.00 | 0.00 ± 0.00 |
| d;Bacteria_p;Firmicutes_c;Bacilli_o;Staphylococcales_f;Gemellaceae_g;Gemella | 0.01 ± 0.02 | 0.01 ± 0.03 |
| d;Bacteria_p;Firmicutes_c;Bacilli_o;Lactobacillales_f;Leuconostocaceae_g;Leuconostoc | 0.00 ± 0.01 | 0.00 ± 0.01 |
| d;Bacteria_p;Patescibacteria_c;Saccharimonadia_o;Saccharimonadales_f;Saccharimonadaceae_g;Saccharimonadaceae | 0.00 ± 0.01 | 0.00 ± 0.02 |
| d;Bacteria_p;Bacteroidota_c;Bacteroidia_o;Bacteroidales_f;Barnesiellaceae_g;Coprobacter | 0.03 ± 0.06 | 0.02 ± 0.05 |
| d;Bacteria_p;Firmicutes_c;Clostridia_o;Lachnospirales_f;uncultured_g;uncultured | 0.00 ± 0.00 | 0.00 ± 0.00 |
| d;Bacteria_p;Proteobacteria_c;Gammaproteobacteria_o;Enterobacterales;_; | 0.01 ± 0.01 | 0.00 ± 0.01 |
| d;Bacteria_p;Proteobacteria_c;Gammaproteobacteria_o;Aeromonadales_f;Aeromonadaceae_g;Aeromonas | 0.00 ± 0.00 | 0.00 ± 0.00 |
| d;Bacteria_p;Firmicutes_c;Clostridia_o;Lachnospirales_f;Lachnospiraceae_g;Howardella | 0.01 ± 0.05 | 0.05 ± 0.14 |
| d;Bacteria_p;Actinobacteriota_c;Actinobacteria_o;Bifidobacteriales_f;Bifidobacteriaceae;_ | 0.00 ± 0.00 | 0.00 ± 0.00 |
| d;Bacteria_p;Firmicutes_c;Bacilli_o;Lactobacillales;_; | 0.00 ± 0.00 | 0.00 ± 0.00 |
| d;Bacteria_p;Proteobacteria_c;Gammaproteobacteria_o;Aeromonadales_f;Succinivibrionaceae_g;Succinatimonas | 0.00 ± 0.02 | 0.01 ± 0.03 |
| d;Bacteria_p;Firmicutes_c;Incertae_Sedis_o;DTU014_f;DTU014_g;DTU014 | 0.00 ± 0.01 | 0.00 ± 0.01 |
| d;Bacteria_p;Firmicutes_c;Clostridia_o;Oscillospirales_f;Ruminococcaceae_g;Candidatus_Soleaferrea | 0.00 ± 0.00 | 0.01 ± 0.02 |
| d;Bacteria_p;Synergistota_c;Synergistia_o;Synergistales_f;Synergistaceae_g;Pyramidobacter | 0.00 ± 0.00 | 0.00 ± 0.00 |
| d;Bacteria_p;Spirochaetota_c;Brachyspirae_o;Brachyspirales_f;Brachyspiraceae_g;Brachyspira | 0.00 ± 0.00 | 0.00 ± 0.01 |
| d;Bacteria_p;Firmicutes_c;Bacilli_o;Erysipelotrichales_f;Erysipelatoclostridiaceae_g;Erysipelatoclostridium | 0.08 ± 0.18 | 0.08 ± 0.21 |
| d;Bacteria_p;Actinobacteriota_c;Coriobacteriia_o;Coriobacteriales_f;Eggerthellaceae_g;Enterorhabdus | 0.00 ± 0.00 | 0.00 ± 0.01 |
| d;Bacteria_p;Desulfobacterota_c;Desulfovibrionia_o;Desulfovibrionales_f;Desulfovibrionaceae_g;uncultured | 0.00 ± 0.00 | 0.00 ± 0.00 |
| d;Bacteria_p;Proteobacteria_c;Gammaproteobacteria_o;Enterobacterales_f;Morganellaceae_g;Proteus | 0.00 ± 0.02 | 0.00 ± 0.01 |
| d;Bacteria_p;Firmicutes_c;Clostridia_o;Peptostreptococcales_Tissierellales_f;Anaerovoracaceae_g;S5_A14a | 0.00 ± 0.00 | 0.00 ± 0.00 |
| d;Bacteria_p;Firmicutes_c;Clostridia_o;Peptostreptococcales_Tissierellales_f;Anaerovoracaceae_g;_Eubacterium;brachy_group | 0.00 ± 0.00 | 0.00 ± 0.00 |
| d;Bacteria_p;Firmicutes_c;Clostridia_o;Christensenellales_f;Christensenellaceae;_ | 0.00 ± 0.01 | 0.00 ± 0.01 |
| d;Bacteria_p;Firmicutes_c;Bacilli_o;Erysipelotrichales_f;Erysipelotrichaceae;_ | 0.00 ± 0.00 | 0.00 ± 0.01 |
| d;Bacteria_p;Actinobacteriota_c;Coriobacteriia_o;Coriobacteriales_f;Atopobiaceae_g;uncultured | 0.00 ± 0.00 | 0.00 ± 0.00 |
| d;Bacteria_p;Firmicutes_c;Clostridia_o;Peptostreptococcales_Tissierellales_f;Anaerovoracaceae;_ | 0.00 ± 0.01 | 0.00 ± 0.01 |
| d;Bacteria_p;Proteobacteria_c;Gammaproteobacteria_o;Aeromonadales_f;Succinivibrionaceae;_ | 0.00 ± 0.00 | 0.00 ± 0.00 |
| d;Bacteria_p;Firmicutes_c;Clostridia_o;Peptostreptococcales_Tissierellales_f;Peptostreptococcales_Tissierellales_g;Parvimonas | 0.00 ± 0.00 | 0.00 ± 0.01 |
| d;Bacteria_p;Proteobacteria_c;Gammaproteobacteria_o;Burkholderiales_f;Oxalobacteraceae;_ | 0.00 ± 0.00 | 0.00 ± 0.02 |
| d;Bacteria_p;Actinobacteriota_c;Actinobacteria_o;Micrococcales_f;Micrococcaceae_g;Rothia | 0.00 ± 0.01 | 0.00 ± 0.01 |
| d;Bacteria_p;Actinobacteriota_c;Actinobacteria;_;__ | 0.00 ± 0.01 | 0.00 ± 0.01 |
| d;Bacteria_p;Actinobacteriota_c;Coriobacteriia_o;Coriobacteriales_f;Eggerthellaceae_g;Eggerthella | 0.00 ± 0.01 | 0.01 ± 0.02 |
| d;Bacteria_p;Firmicutes_c;Bacilli_o;Acholeplasmatales_f;Acholeplasmataceae_g;Anaeroplasma | 0.00 ± 0.02 | 0.00 ± 0.00 |
| d;Bacteria_p;Actinobacteriota_c;Coriobacteriia_o;Coriobacteriales;_; | 0.00 ± 0.00 | 0.00 ± 0.00 |
| d;Bacteria_p;Proteobacteria_c;Alphaproteobacteria_o;Sphingomonadales_f;Sphingomonadaceae;_ | 0.00 ± 0.00 | 0.00 ± 0.00 |
| d;Bacteria_p;Firmicutes_c;Clostridia_o;Lachnospirales_f;Lachnospiraceae_g;Cellulosilyticum | 0.00 ± 0.00 | 0.00 ± 0.00 |
| d;Bacteria_p;Synergistota_c;Synergistia_o;Synergistales_f;Synergistaceae_g;Synergistes | 0.00 ± 0.00 | 0.00 ± 0.00 |
| d;Bacteria_p;Actinobacteriota_c;Coriobacteriia_o;Coriobacteriales_f;Eggerthellaceae_g;Parvibacter | 0.00 ± 0.00 | 0.00 ± 0.00 |
| d;Bacteria_p;Firmicutes_c;Bacilli_o;Erysipelotrichales;_; | 0.00 ± 0.00 | 0.00 ± 0.00 |
| d;Bacteria_p;Firmicutes_c;Clostridia_o;Lachnospirales_f;Lachnospiraceae_g;FD2005 | 0.00 ± 0.00 | 0.00 ± 0.00 |
| d;Bacteria_p;Firmicutes_c;Clostridia_o;Eubacteriales_f;Eubacteriaceae_g;Eubacterium | 0.00 ± 0.00 | 0.00 ± 0.00 |
| d;Bacteria_p;Firmicutes_c;Clostridia_o;Eubacteriales_f;Eubacteriaceae;_ | 0.00 ± 0.00 | 0.00 ± 0.00 |
| d;Bacteria_p;Firmicutes_c;Clostridia_o;Lachnospirales_f;Lachnospiraceae_g;Lachnospiraceae_UCG_003 | 0.01 ± 0.04 | 0.00 ± 0.01 |
| d;Bacteria_p;Patescibacteria_c;Saccharimonadia_o;Saccharimonadales_f;Saccharimonadaceae_g;TM7x | 0.02 ± 0.05 | 0.02 ± 0.03 |
| d;Bacteria_p;Bacteroidota_c;Bacteroidia_o;Bacteroidales_f;Prevotellaceae_g;Prevotellaceae_UCG_001 | 0.00 ± 0.00 | 0.00 ± 0.02 |
| d;Bacteria_p;Firmicutes_c;Bacilli_o;Erysipelotrichales_f;Erysipelotrichaceae_g;Solobacterium | 0.01 ± 0.02 | 0.04 ± 0.12 |
| d;Bacteria_p;Firmicutes_c;Clostridia_o;Lachnospirales_f;Lachnospiraceae_g;_Eubacterium;xylanophilum_group | 0.05 ± 0.12 | 0.02 ± 0.04 |
| d;Bacteria_p;Firmicutes_c;Clostridia_o;Lachnospirales_f;Lachnospiraceae_g;Pseudobutyrivibrio | 0.00 ± 0.01 | 0.00 ± 0.01 |
| d;Bacteria_p;Firmicutes_c;Clostridia_o;uncultured_f;uncultured_g;uncultured | 0.00 ± 0.00 | 0.00 ± 0.00 |
| d;Bacteria_p;Firmicutes_c;Bacilli;_ | 0.00 ± 0.00 | 0.00 ± 0.00 |
| d;Bacteria_p;Proteobacteria_c;Gammaproteobacteria_o;Pseudomonadales_f;Pseudomonadaceae_g;Pseudomonas | 0.00 ± 0.00 | 0.00 ± 0.00 |
| d;Bacteria_p;Actinobacteriota_c;Coriobacteriia_o;Coriobacteriales_f;Eggerthellaceae_g;Paraeggerthella | 0.00 ± 0.00 | 0.00 ± 0.00 |
| d;Bacteria_p;Firmicutes_c;Bacilli_o;Lactobacillales_f;Lactobacillaceae_g;Pediococcus | 0.00 ± 0.00 | 0.00 ± 0.01 |
| d;Bacteria_p;Firmicutes_c;Clostridia_o;Clostridiales_f;Clostridiaceae;_ | 0.00 ± 0.00 | 0.00 ± 0.01 |
| d;Bacteria_p;Elusimicrobiota_c;Elusimicrobia_o;Elusimicrobiales_f;Elusimicrobiaceae_g;Elusimicrobium | 0.00 ± 0.01 | 0.02 ± 0.08 |
| d;Bacteria_p;Actinobacteriota_c;Coriobacteriia_o;Coriobacteriales_f;Eggerthellaceae_g;DNF00809 | 0.00 ± 0.00 | 0.00 ± 0.00 |
| d;Bacteria_p;Bacteroidota_c;Bacteroidia_o;Bacteroidales_f;Barnesiellaceae_g;uncultured | 0.00 ± 0.00 | 0.00 ± 0.00 |
| d;Bacteria_p;Firmicutes_c;Clostridia_o;Oscillospirales_f;UCG_011_g;UCG_011 | 0.00 ± 0.00 | 0.00 ± 0.01 |
| d;Bacteria_p;Firmicutes_c;Clostridia_o;Peptostreptococcales_Tissierellales_f;Anaerovoracaceae_g;_Eubacterium;nodatum_group | 0.00 ± 0.01 | 0.01 ± 0.02 |
| d;Bacteria_p;Firmicutes_c;Bacilli_o;Erysipelotrichales_f;Erysipelotrichaceae_g;Merdibacter | 0.00 ± 0.00 | 0.00 ± 0.01 |
| d;Bacteria_p;Firmicutes_c;Clostridia_o;Oscillospirales_f;Oscillospiraceae_g;Papillibacter | 0.00 ± 0.00 | 0.00 ± 0.01 |
| d;Bacteria_p;Deferribacterota_c;Deferribacteres_o;Deferribacterales_f;Deferribacteraceae_g;Mucispirillum | 0.00 ± 0.00 | 0.00 ± 0.00 |
| d;Bacteria_p;Firmicutes_c;Clostridia_o;Peptostreptococcales_Tissierellales_f;Peptostreptococcaceae_g;Terrisporobacter | 0.00 ± 0.00 | 0.00 ± 0.00 |
| d;Bacteria_p;Proteobacteria_c;Gammaproteobacteria_o;Pasteurellales_f;Pasteurellaceae_g;Actinobacillus | 0.00 ± 0.00 | 0.00 ± 0.00 |
| d;Bacteria_p;Firmicutes_c;Bacilli_o;Erysipelotrichales_f;Erysipelatoclostridiaceae_g;Erysipelatoclostridiaceae | 0.00 ± 0.00 | 0.00 ± 0.01 |
| d;Bacteria_p;Firmicutes_c;Clostridia_o;Lachnospirales_f;Lachnospiraceae_g;GCA_900066575 | 0.02 ± 0.03 | 0.02 ± 0.02 |
| d;Bacteria_p;Proteobacteria_c;Gammaproteobacteria_o;Enterobacterales_f;Enterobacteriaceae_g;Citrobacter | 0.00 ± 0.00 | 0.00 ± 0.00 |
| d;Bacteria_p;Firmicutes_c;Clostridia_o;Lachnospirales_f;Lachnospiraceae_g;Lactonifactor | 0.00 ± 0.01 | 0.00 ± 0.00 |
| d;Bacteria_p;Firmicutes_c;Clostridia_o;Lachnospirales_f;Lachnospiraceae_g;Lachnospiraceae_AC2044_group | 0.00 ± 0.00 | 0.00 ± 0.00 |
| d;Bacteria_p;Firmicutes_c;Bacilli_o;Erysipelotrichales_f;Erysipelatoclostridiaceae;_ | 0.01 ± 0.02 | 0.00 ± 0.01 |
| d;Bacteria_p;Proteobacteria_c;Gammaproteobacteria_o;Aeromonadales;_; | 0.00 ± 0.00 | 0.00 ± 0.00 |
| d;Bacteria_p;Firmicutes_c;Clostridia_o;Lachnospirales_f;Lachnospiraceae_g;Lachnospiraceae_UCG_008 | 0.00 ± 0.00 | 0.00 ± 0.01 |
| d;Bacteria_p;Bacteroidota_c;Bacteroidia_o;Bacteroidales_f;Muribaculaceae_g;CAG_873 | 0.00 ± 0.00 | 0.00 ± 0.00 |
| d;Bacteria_p;Firmicutes_c;Negativicutes_o;Veillonellales_Selenomonadales_f;Veillonellaceae;_ | 0.00 ± 0.00 | 0.00 ± 0.00 |
| d;Bacteria_p;Firmicutes_c;Clostridia_o;Oscillospirales_f;Ruminococcaceae_g;Caproiciproducens | 0.00 ± 0.00 | 0.00 ± 0.00 |
| d;Bacteria_p;Firmicutes_c;Clostridia_o;Oscillospirales_f;Oscillospirales_g;Hydrogenoanaerobacterium | 0.00 ± 0.02 | 0.00 ± 0.00 |
| d;Bacteria_p;Bacteroidota_c;Bacteroidia_o;Bacteroidales_f;Marinifilaceae_g;Sanguibacteroides | 0.00 ± 0.00 | 0.00 ± 0.02 |
| d;Bacteria_p;Firmicutes_c;Clostridia_o;Lachnospirales_f;Lachnospiraceae_g;_Ruminococcus;gnavus_group | 0.72 ± 2.47 | 0.18 ± 0.42 |
| d;Bacteria_p;Acidobacteriota_c;Blastocatellia_o;Blastocatellales_f;Blastocatellaceae;_ | 0.00 ± 0.00 | 0.00 ± 0.00 |
| d;Bacteria_p;Firmicutes_c;Clostridia_o;Lachnospirales_f;Lachnospiraceae_g;GCA_900066755 | 0.00 ± 0.00 | 0.00 ± 0.01 |
| d;Bacteria_p;Firmicutes_c;Clostridia_o;Clostridia_f;Hungateiclostridiaceae;_ | 0.00 ± 0.00 | 0.00 ± 0.00 |
| d;Bacteria_p;Actinobacteriota_c;Actinobacteria_o;Actinomycetales_f;Actinomycetaceae_g;Actinotignum | 0.00 ± 0.00 | 0.00 ± 0.00 |
| d;Bacteria_p;Bacteroidota_c;Bacteroidia_o;Bacteroidales_f;Marinifilaceae;_ | 0.00 ± 0.00 | 0.00 ± 0.00 |
| d;Bacteria_p;Proteobacteria_c;Alphaproteobacteria_o;Rhizobiales_f;Xanthobacteraceae_g;Bradyrhizobium | 0.00 ± 0.00 | 0.00 ± 0.00 |
| d;Bacteria_p;Proteobacteria_c;Alphaproteobacteria;_ | 0.01 ± 0.04 | 0.00 ± 0.01 |
| d;Bacteria_p;Firmicutes_c;Bacilli_o;Erysipelotrichales_f;Erysipelotrichaceae_g;Erysipelotrichaceae | 0.00 ± 0.00 | 0.00 ± 0.00 |
| d;Bacteria_p;Desulfobacterota_c;Desulfovibrionia_o;Desulfovibrionales_f;Desulfovibrionaceae_g;Mailhella | 0.00 ± 0.01 | 0.00 ± 0.00 |
| d;Bacteria_p;Firmicutes_c;Bacilli_o;Erysipelotrichales_f;Erysipelotrichaceae_g;Catenisphaera | 0.00 ± 0.00 | 0.00 ± 0.00 |
| d;Bacteria_p;Firmicutes_c;Clostridia_o;Lachnospirales_f;Lachnospiraceae_g;_Bacteroides;pectinophilus_group | 0.00 ± 0.00 | 0.00 ± 0.00 |
| d;Bacteria_p;Firmicutes_c;Clostridia_o;Oscillospirales_f;Ethanoligenenaceae_g;Acetanaerobacterium | 0.00 ± 0.01 | 0.00 ± 0.01 |
| d;Bacteria_p;Proteobacteria_c;Gammaproteobacteria_o;Burkholderiales_f;Neisseriaceae_g;Eikenella | 0.00 ± 0.00 | 0.00 ± 0.00 |
| d;Bacteria_p;Firmicutes_c;Clostridia_o;Clostridia_f;Hungateiclostridiaceae_g;Ruminiclostridium | 0.00 ± 0.00 | 0.00 ± 0.00 |
| d;Bacteria_p;Actinobacteriota_c;Coriobacteriia_o;Coriobacteriales_f;Atopobiaceae_g;Coriobacteriaceae_UCG_003 | 0.00 ± 0.00 | 0.00 ± 0.02 |
| d;Bacteria_p;Firmicutes_c;Clostridia_o;Lachnospirales_f;Lachnospiraceae_g;Lachnospiraceae_ND3007_group | 0.03 ± 0.05 | 0.06 ± 0.10 |
| d;Bacteria_p;Firmicutes_c;Clostridia_o;Peptostreptococcales_Tissierellales_f;Peptostreptococcales_Tissierellales_g;Ezakiella | 0.00 ± 0.00 | 0.00 ± 0.01 |
| d;Bacteria_p;Bacteroidota_c;Bacteroidia_o;Bacteroidales_f;Prevotellaceae_g;Prevotellaceae_UCG_003 | 0.13 ± 0.66 | 0.03 ± 0.11 |
| d;Bacteria_p;Proteobacteria_c;Gammaproteobacteria_o;Burkholderiales_f;Burkholderiaceae_g;Lautropia | 0.00 ± 0.00 | 0.00 ± 0.00 |
| d;Bacteria_p;Actinobacteriota_c;Coriobacteriia_o;Coriobacteriales_f;Coriobacteriales_Incertae_Sedis_g;Raoultibacter | 0.00 ± 0.01 | 0.00 ± 0.01 |
| d;Bacteria_p;Proteobacteria_c;Gammaproteobacteria_o;Enterobacterales_f;Yersiniaceae_g;Serratia | 0.00 ± 0.00 | 0.00 ± 0.00 |
| d;Bacteria_p;Proteobacteria_c;Gammaproteobacteria_o;Enterobacterales_f;Enterobacteriaceae_g;Franconibacter | 0.00 ± 0.00 | 0.00 ± 0.00 |
| d;Bacteria_p;Firmicutes_c;Negativicutes_o;Veillonellales_Selenomonadales_f;Selenomonadaceae_g;uncultured | 0.00 ± 0.00 | 0.00 ± 0.02 |
| d;Bacteria_p;Proteobacteria_c;Gammaproteobacteria_o;Burkholderiales_f;Comamonadaceae_g;Delftia | 0.00 ± 0.00 | 0.00 ± 0.00 |
| d;Bacteria_p;Firmicutes_c;Clostridia_o;Lachnospirales_f;Lachnospiraceae_g;_Eubacterium;fissicatena_group | 0.00 ± 0.00 | 0.00 ± 0.01 |
| d;Bacteria_p;Firmicutes_c;Clostridia_o;Oscillospirales_f;Oscillospiraceae_g;Pseudoflavonifractor | 0.00 ± 0.00 | 0.00 ± 0.01 |
| d;Bacteria_p;Firmicutes_c;Negativicutes_o;Veillonellales_Selenomonadales_f;Veillonellaceae_g;Anaeroglobus | 0.00 ± 0.00 | 0.00 ± 0.00 |
| d;Bacteria_p;Actinobacteriota_c;Coriobacteriia_o;Coriobacteriales_f;Atopobiaceae_g;Atopobium | 0.01 ± 0.01 | 0.00 ± 0.00 |
| d;Bacteria_p;Firmicutes_c;Clostridia_o;Peptostreptococcales_Tissierellales_f;Anaerovoracaceae_g;_Eubacterium;saphenum_group | 0.00 ± 0.00 | 0.00 ± 0.00 |
| d;Bacteria_p;Firmicutes_c;Clostridia_o;Lachnospirales_f;Lachnospiraceae_g;UC5_1_2E3 | 0.00 ± 0.00 | 0.01 ± 0.03 |
| d;Bacteria_p;Firmicutes_c;Clostridia_o;Peptostreptococcales_Tissierellales_f;Peptostreptococcaceae_g;Intestinibacter | 0.01 ± 0.03 | 0.02 ± 0.03 |
| d;Bacteria_p;Firmicutes_c;Bacilli_o;Erysipelotrichales_f;Erysipelotrichaceae_g;uncultured | 0.00 ± 0.00 | 0.00 ± 0.00 |
| d;Bacteria_p;Chloroflexi_c;Anaerolineae_o;Anaerolineales_f;Anaerolineaceae_g;Flexilinea | 0.00 ± 0.00 | 0.00 ± 0.00 |
| d;Bacteria_p;Actinobacteriota_c;Actinobacteria_o;Bifidobacteriales_f;Bifidobacteriaceae_g;Scardovia | 0.00 ± 0.00 | 0.00 ± 0.00 |
| d;Bacteria_p;Firmicutes_c;Bacilli_o;Izemoplasmatales_f;Izemoplasmatales_g;Izemoplasmatales | 0.01 ± 0.06 | 0.03 ± 0.11 |
| d;Bacteria_p;Actinobacteriota_c;Actinobacteria_o;Actinomycetales_f;Actinomycetaceae_g;F0332 | 0.00 ± 0.00 | 0.00 ± 0.00 |
| d;Bacteria_p;Firmicutes_c;Clostridia_o;Peptostreptococcales_Tissierellales;_; | 0.00 ± 0.00 | 0.00 ± 0.00 |
| d;Bacteria_p;Proteobacteria_c;Alphaproteobacteria_o;Rhodobacterales_f;Rhodobacteraceae;_ | 0.00 ± 0.00 | 0.00 ± 0.00 |
| d;Bacteria_p;Firmicutes_c;Clostridia_o;Lachnospirales_f;Lachnospiraceae_g;Lachnoanaerobaculum | 0.00 ± 0.00 | 0.00 ± 0.01 |
| d;Bacteria_p;Actinobacteriota_c;Coriobacteriia_o;Coriobacteriales_f;Atopobiaceae;_ | 0.00 ± 0.00 | 0.10 ± 0.49 |
| d;Bacteria_p;Firmicutes_c;Clostridia_o;Lachnospirales_f;Lachnospiraceae_g;CHKCI001 | 0.00 ± 0.00 | 0.00 ± 0.00 |
| d;Bacteria_p;Proteobacteria_c;Alphaproteobacteria_o;Rhizobiales_f;Rhizobiaceae;_ | 0.00 ± 0.00 | 0.00 ± 0.00 |
| d;Bacteria_p;Firmicutes_c;Clostridia_o;Lachnospirales_f;Lachnospiraceae_g;Anaerostignum | 0.00 ± 0.02 | 0.00 ± 0.00 |
| d;Bacteria_p;Firmicutes_c;Clostridia_o;Lachnospirales_f;Lachnospiraceae_g;_Ruminococcus;gauvreauii_group | 0.01 ± 0.03 | 0.02 ± 0.08 |
| d;Bacteria_p;Firmicutes_c;Bacilli_o;Erysipelotrichales_f;Erysipelatoclostridiaceae_g;UCG_004 | 0.00 ± 0.01 | 0.00 ± 0.01 |
| d;Bacteria_p;Firmicutes_c;Bacilli_o;Erysipelotrichales_f;Erysipelotrichaceae_g;Faecalicoccus | 0.00 ± 0.00 | 0.00 ± 0.00 |
| d;Bacteria_p;Firmicutes_c;Clostridia_o;Peptostreptococcales_Tissierellales_f;Peptostreptococcaceae_g;Peptostreptococcus | 0.00 ± 0.02 | 0.00 ± 0.00 |
| d;Bacteria_p;Proteobacteria_c;Gammaproteobacteria_o;Burkholderiales_f;Comamonadaceae;_ | 0.00 ± 0.00 | 0.00 ± 0.00 |
| d;Bacteria_p;Firmicutes_c;Clostridia_o;Oscillospirales_f;Butyricicoccaceae_g;uncultured | 0.03 ± 0.13 | 0.00 ± 0.00 |
| d;Bacteria_p;Campilobacterota_c;Campylobacteria_o;Campylobacterales_f;Helicobacteraceae_g;Helicobacter | 0.00 ± 0.00 | 0.00 ± 0.00 |
| d;Bacteria_p;Fusobacteriota_c;Fusobacteriia_o;Fusobacteriales_f;Fusobacteriaceae_g;Cetobacterium | 0.00 ± 0.00 | 0.00 ± 0.00 |
| d;Bacteria_p;Firmicutes_c;Clostridia_o;Peptostreptococcales_Tissierellales_f;Anaerovoracaceae_g;Mogibacterium | 0.00 ± 0.00 | 0.00 ± 0.00 |
| d;Bacteria_p;Firmicutes_c;Clostridia_o;Peptostreptococcales_Tissierellales_f;Peptostreptococcaceae_g;Clostridioides | 0.00 ± 0.01 | 0.00 ± 0.00 |
| d;Bacteria_p;Proteobacteria_c;Gammaproteobacteria_o;Enterobacterales_f;Pectobacteriaceae_g;Dickeya | 0.00 ± 0.00 | 0.00 ± 0.00 |
| d;Bacteria_p;Firmicutes_c;Bacilli_o;Lactobacillales_f;Aerococcaceae_g;Aerococcus | 0.00 ± 0.00 | 0.00 ± 0.00 |
| d;Bacteria_p;Bacteroidota_c;Bacteroidia_o;Bacteroidales_f;Dysgonomonadaceae_g;Proteiniphilum | 0.00 ± 0.00 | 0.00 ± 0.00 |
| d;Bacteria_p;Proteobacteria_c;Gammaproteobacteria_o;Burkholderiales_f;Methylophilaceae_g;Methylophilus | 0.00 ± 0.00 | 0.00 ± 0.00 |
| d;Bacteria_p;Firmicutes_c;Clostridia_o;Clostridiales_f;Clostridiaceae_g;Clostridium_sensu_stricto_2 | 0.00 ± 0.00 | 0.00 ± 0.00 |
| d;Bacteria_p;Firmicutes_c;Negativicutes_o;Veillonellales_Selenomonadales_f;Veillonellaceae_g;uncultured | 0.00 ± 0.00 | 0.00 ± 0.00 |
| d;Bacteria_p;Firmicutes_c;Clostridia_o;Lachnospirales_f;Lachnospiraceae_g;Shuttleworthia | 0.00 ± 0.00 | 0.00 ± 0.00 |
| d;Bacteria_p;Proteobacteria_c;Gammaproteobacteria_o;Burkholderiales_f;Sutterellaceae;_ | 0.00 ± 0.00 | 0.00 ± 0.00 |
| d;Bacteria_p;Bacteroidota_c;Bacteroidia_o;Flavobacteriales_f;Flavobacteriaceae_g;Flavobacterium | 0.00 ± 0.00 | 0.00 ± 0.00 |
| d;Bacteria_p;Verrucomicrobiota_c;Verrucomicrobiae_o;Opitutales_f;Puniceicoccaceae;_ | 0.00 ± 0.00 | 0.00 ± 0.00 |
| d;Bacteria_p;Bacteroidota_c;Bacteroidia_o;Bacteroidales_f;Prevotellaceae_g;Prevotellaceae | 0.00 ± 0.00 | 0.00 ± 0.00 |
| d;Bacteria_p;Firmicutes_c;Clostridia_o;Oscillospirales_f;Oscillospiraceae_g;UCG_007 | 0.00 ± 0.00 | 0.00 ± 0.00 |
| d;Bacteria_p;Cyanobacteria_c;Vampirivibrionia_o;Vampirovibrionales_f;Vampirovibrionales_g;Vampirovibrionales | 0.00 ± 0.00 | 0.00 ± 0.00 |
| d;Bacteria_p;Bacteroidota_c;Bacteroidia_o;Bacteroidales_f;Rikenellaceae_g;SP3_e08 | 0.00 ± 0.00 | 0.00 ± 0.00 |
| d;Bacteria_p;Actinobacteriota_c;Actinobacteria_o;Bifidobacteriales_f;Bifidobacteriaceae_g;Alloscardovia | 0.00 ± 0.00 | 0.00 ± 0.00 |
| d;Bacteria_p;Firmicutes_c;Bacilli_o;Lactobacillales_f;Aerococcaceae_g;Facklamia | 0.00 ± 0.00 | 0.00 ± 0.00 |
| d;Bacteria_p;Firmicutes_c;Clostridia_o;Clostridiales;_; | 0.00 ± 0.00 | 0.00 ± 0.00 |
| d;Bacteria_p;Firmicutes_c;Negativicutes;_;__ | 0.00 ± 0.00 | 0.00 ± 0.00 |
| d;Bacteria_p;Actinobacteriota_c;Actinobacteria_o;Micrococcales;_; | 0.00 ± 0.00 | 0.00 ± 0.00 |
| d;Bacteria_p;Actinobacteriota_c;Actinobacteria_o;Actinomycetales_f;Actinomycetaceae_g;Varibaculum | 0.00 ± 0.00 | 0.00 ± 0.00 |
| d;Bacteria_p;Firmicutes_c;Bacilli_o;Mycoplasmatales_f;Mycoplasmataceae_g;Mycoplasma | 0.00 ± 0.00 | 0.00 ± 0.00 |
| d;Bacteria_p;Bacteroidota_c;Bacteroidia_o;Bacteroidales_f;Rikenellaceae_g;Rikenella | 0.00 ± 0.00 | 0.00 ± 0.00 |
| d;Bacteria_p;Firmicutes_c;Bacilli_o;Mycoplasmatales_f;Mycoplasmataceae_g;Ureaplasma | 0.00 ± 0.00 | 0.00 ± 0.00 |
| d;Bacteria_p;Firmicutes_c;Clostridia_o;Lachnospirales_f;Lachnospiraceae_g;Epulopiscium | 0.00 ± 0.00 | 0.00 ± 0.00 |
| d;Bacteria_p;Actinobacteriota_c;Actinobacteria_o;Corynebacteriales_f;Mycobacteriaceae_g;Mycobacterium | 0.00 ± 0.00 | 0.00 ± 0.00 |
| d;Bacteria_p;Firmicutes_c;Bacilli_o;Staphylococcales_f;Staphylococcaceae_g;Staphylococcus | 0.00 ± 0.00 | 0.00 ± 0.00 |
| d;Bacteria_p;Desulfobacterota_c;Desulfovibrionia_o;Desulfovibrionales;_ | 0.00 ± 0.00 | 0.00 ± 0.00 |
| d;Bacteria_p;Actinobacteriota_c;Coriobacteriia_o;Coriobacteriales_f;Eggerthellaceae_g;CHKCI002 | 0.00 ± 0.00 | 0.00 ± 0.00 |
| d;Bacteria_p;Actinobacteriota_c;Actinobacteria_o;Corynebacteriales_f;Corynebacteriaceae_g;Lawsonella | 0.00 ± 0.00 | 0.00 ± 0.00 |
| d;Bacteria_p;Firmicutes_c;Bacilli_o;Lactobacillales_f;Aerococcaceae_g;Abiotrophia | 0.00 ± 0.02 | 0.00 ± 0.01 |
| d;Bacteria_p;Proteobacteria_c;Gammaproteobacteria_o;Enterobacterales_f;Erwiniaceae;_ | 0.00 ± 0.00 | 0.00 ± 0.00 |
| d;Bacteria_p;Firmicutes_c;Clostridia_o;Lachnospirales_f;Lachnospiraceae_g;CAG_56 | 0.00 ± 0.00 | 0.00 ± 0.00 |
| d;Bacteria_p;Firmicutes_c;Clostridia_o;Clostridiales_f;Clostridiaceae_g;Candidatus_Arthromitus | 0.00 ± 0.00 | 0.00 ± 0.00 |
| d;Bacteria_p;Firmicutes_c;Clostridia_o;Clostridiales_f;Clostridiaceae_g;Clostridium_sensu_stricto_3 | 0.00 ± 0.00 | 0.00 ± 0.00 |
| Unassigned;_ | 0.00 ± 0.00 | 0.00 ± 0.00 |
| d;Bacteria_p;Proteobacteria_c;Gammaproteobacteria_o;Aeromonadales_f;Succinivibrionaceae_g;uncultured | 0.00 ± 0.00 | 0.00 ± 0.00 |
| d;Bacteria_p;Actinobacteriota_c;Coriobacteriia_o;Coriobacteriales_f;Coriobacteriaceae_g;Enorma | 0.00 ± 0.00 | 0.00 ± 0.00 |
| d;Bacteria_p;Actinobacteriota_c;Actinobacteria_o;Actinomycetales_f;Actinomycetaceae_g;Mobiluncus | 0.00 ± 0.00 | 0.00 ± 0.00 |
| d;Bacteria_p;Proteobacteria_c;Gammaproteobacteria_o;Enterobacterales_f;Morganellaceae_g;Providencia | 0.00 ± 0.00 | 0.00 ± 0.00 |
| d;Bacteria_p;Proteobacteria_c;Gammaproteobacteria_o;Burkholderiales_f;Burkholderiaceae_g;Burkholderia_Caballeronia_Paraburkholderia | 0.00 ± 0.00 | 0.00 ± 0.00 |
| d;Bacteria_p;Proteobacteria_c;Alphaproteobacteria_o;Rhizobiales_f;Labraceae_g;Labrys | 0.00 ± 0.00 | 0.00 ± 0.00 |
| d;Bacteria_p;Firmicutes_c;Clostridia_o;Lachnospirales_f;Lachnospiraceae_g;Lachnospiraceae_NK3A20_group | 0.00 ± 0.00 | 0.00 ± 0.00 |
| d;Bacteria_p;Firmicutes_c;Clostridia_o;Lachnospirales_f;Lachnospiraceae_g;Stomatobaculum | 0.00 ± 0.00 | 0.00 ± 0.00 |
| d;Bacteria_p;Actinobacteriota_c;Actinobacteria_o;Corynebacteriales_f;Corynebacteriaceae_g;Corynebacterium | 0.00 ± 0.00 | 0.00 ± 0.00 |
| d;Bacteria_p;Firmicutes_c;Clostridia_o;Peptostreptococcales_Tissierellales_f;Peptostreptococcales_Tissierellales_g;Murdochiella | 0.00 ± 0.00 | 0.00 ± 0.00 |
| d;Bacteria_p;Bacteroidota_c;Bacteroidia_o;Bacteroidales_f;Rikenellaceae;_ | 0.00 ± 0.00 | 0.00 ± 0.00 |
| d;Bacteria_p;Firmicutes_c;Clostridia_o;Oscillospirales_f;Oscillospiraceae_g;V9D2013_group | 0.00 ± 0.00 | 0.00 ± 0.00 |
| d;Bacteria_p;Firmicutes_c;Bacilli_o;Lactobacillales_f;P5D1_392_g;P5D1_392 | 0.00 ± 0.00 | 0.00 ± 0.00 |
| d;Bacteria_p;Proteobacteria_c;Gammaproteobacteria_o;Aeromonadales_f;Aeromonadaceae_g;Tolumonas | 0.00 ± 0.00 | 0.00 ± 0.00 |
| d;Bacteria_p;Proteobacteria_c;Gammaproteobacteria_o;Salinisphaerales_f;Solimonadaceae_g;Nevskia | 0.00 ± 0.00 | 0.00 ± 0.00 |
| d;Bacteria_p;Actinobacteriota_c;Coriobacteriia_o;Coriobacteriales_f;Eggerthellaceae_g;Cryptobacterium | 0.00 ± 0.00 | 0.00 ± 0.00 |
| d;Bacteria_p;Firmicutes_c;Negativicutes_o;Veillonellales_Selenomonadales;_; | 0.00 ± 0.00 | 0.00 ± 0.00 |
| d;Bacteria_p;Firmicutes_c;Clostridia_o;Clostridia_f;Hungateiclostridiaceae_g;Saccharofermentans | 0.00 ± 0.00 | 0.00 ± 0.00 |
| d;Bacteria_p;Desulfobacterota_c;Desulfobulbia_o;Desulfobulbales_f;Desulfurivibrionaceae_g;Desulfurivibrio | 0.00 ± 0.00 | 0.00 ± 0.00 |
| d;Bacteria_p;Firmicutes_c;Clostridia_o;Oscillospirales_f;Oscillospiraceae_g;Oscillospiraceae | 0.00 ± 0.00 | 0.00 ± 0.00 |
| d;Bacteria_p;Firmicutes_c;Clostridia_o;Peptostreptococcales_Tissierellales_f;Peptostreptococcales_Tissierellales_g;W5053 | 0.00 ± 0.00 | 0.00 ± 0.00 |
| d;Bacteria_p;Proteobacteria_c;Alphaproteobacteria_o;Rhizobiales_f;Beijerinckiaceae_g;Methylobacterium_Methylorubrum | 0.00 ± 0.00 | 0.00 ± 0.00 |
| d;Bacteria_p;Proteobacteria_c;Alphaproteobacteria_o;Sphingomonadales_f;Sphingomonadaceae_g;Sphingobium | 0.00 ± 0.00 | 0.00 ± 0.00 |
| d;Bacteria_p;Bacteroidota_c;Bacteroidia_o;Bacteroidales_f;Marinifilaceae_g;uncultured | 0.00 ± 0.00 | 0.00 ± 0.00 |

d; domain, p; phylum, c; class, o; order, f; family, g; genus

Data are expressed as mean ± standard deviation
